# Identification of Position-Specific Correlations between DNA-Binding Domains and Their Binding Sites. Application to the MerR Family of Transcription Factors

**DOI:** 10.1371/journal.pone.0162681

**Published:** 2016-09-30

**Authors:** Yuriy D. Korostelev, Ilya A. Zharov, Andrey A. Mironov, Alexandra B. Rakhmaininova, Mikhail S. Gelfand

**Affiliations:** 1 A.A. Kharkevich Institute for Information Transmission Problems, Russian Academy of Sciences, 19-1 Bolshoy Karetny pereulok, Moscow, Russia, 127994; 2 Department of Bioengineering and Bioinformatics, Moscow State University, 1-73 Vorobievy Gory, Moscow, Russia, 119991; University of North Carolina at Charlotte, UNITED STATES

## Abstract

The large and increasing volume of genomic data analyzed by comparative methods provides information about transcription factors and their binding sites that, in turn, enables statistical analysis of correlations between factors and sites, uncovering mechanisms and evolution of specific protein-DNA recognition. Here we present an online tool, Prot-DNA-Korr, designed to identify and analyze crucial protein-DNA pairs of positions in a family of transcription factors. Correlations are identified by analysis of mutual information between columns of protein and DNA alignments. The algorithm reduces the effects of common phylogenetic history and of abundance of closely related proteins and binding sites. We apply it to five closely related subfamilies of the MerR family of bacterial transcription factors that regulate heavy metal resistance systems. We validate the approach using known 3D structures of MerR-family proteins in complexes with their cognate DNA binding sites and demonstrate that a significant fraction of correlated positions indeed form specific side-chain-to-base contacts. The joint distribution of amino acids and nucleotides hence may be used to predict changes of specificity for point mutations in transcription factors.

## Introduction

Specific binding of transcription factors to DNA is a major mechanism of regulation of gene expression, hence boosting interest to the problem of the protein-DNA recognition code. Initial hopes stemmed from the observations that single amino acid substitutions can drastically change the protein affinity to its DNA sites. On the other hand, the structure of the DNA double helix is relatively rigid. An early (mid-70s) paper suggested that specific recognition depends on hydrogen bonds between side chains of amino acid residues and nucleotides bases, demonstrated that this recognition is easier in the major groove of the double helix than in the minor one, and discussed the role of the guanidine group of arginine in the recognition of the GC base pair [1].

The substantial progress in the 80s and 90s was based on the analysis of X-ray structures of protein-DNA complexes. It has been established that the recognition depends not only on hydrogen bonds, but on other types of weak interactions, and some empirical rules of the protein-DNA recognition have been suggested. Analysis of twenty structures demonstrated that the most common contacts between amino acid residues and nucleotide bases may be explained by the physical and chemical properties of the residues—the hydrophobic methyl group of alanine often interacts with the methyl group of thymine; arginine forms two hydrogen bonds with guanine; asparagine forms two hydrogen with adenine; etc. [2]. Moreover, while the orientation of DNA-binding protein structural elements varies in different protein families, within a family the binding is defined by a fixed, limited set of positions. For example, in the helix-turn-helix (HTH) domains, the binding element is the second *α*-helix, with residues 1, 2, 6 recognizing four successive bases in the major groove [2].

These rules were subsequently confirmed in a larger study that analyzed 129 protein-DNA complexes with close homologs filtered out [3]. About one third of ‘residue side chain—base’ hydrogen bonds are involved in complex interactions where one residue interacts with two consecutive nucleotides in DNA. In addition to universal contacts, there exist context-dependent contacts contributing to the recognition specificity, but unique for a given complex.

Currently it is widely accepted that, unlike protein-protein contacts, the regions of protein-DNA contacts are rich in polar residues (Arg, Ser, Tyr, Thr, Asn) [4]. The most positively charged patch on the protein surface often coincides with the DNA-binding site. Purines are more selective in their contacts than pyrimidines [4]. Aromatic amino acids have different specificities, e.g. phenylalanine prefers adenine and thymine, and histidine prefers thymine and guanine [5].

At the same time, there are as many exceptions as there are rules [6]. There is no simple relationship between the amino acid sequence of a protein and the nucleotide sequence of its binding DNA site, and the protein-DNA code is degenerate on both sides [7]. This is not surprising, given the existence of complex contacts [2, 8] and diversity of contact geometries and docking surfaces [9], even for structurally similar proteins [10]. Moreover, the protein’s interaction with its sites is not an all-or-nothing, but rather a quantitative parameter [11], not limited to the chemical identity of the interacting residues and bases, but involving changes in the protein and/or DNA conformation upon interaction known as indirect readout [6]. This shifted the focus of attention from identification of empirical rules to creation of statistical functions based on structural data [6] using neural networks [12, 13], support vector machines [13], or Bayesian classifiers [14] trained on known structures and then applied to protein sequences.

High-throughput experimental techniques such as SELEX [15], ChIP-chip [16, 17], DIP-ChIP [18], ChIP-Seq [19] and PBMs [20], as well as comparative genomic analyses [21, 22] provide large number of binding sites for a given TF. Available data on binding sites of transcription factors, collected in databases such as TRANSFAC [23], JASPAR [24], Factorbook [25], RegTransBase [26], and RegPrecise [27], exceed by orders of magnitude the number of solved structures of protein-DNA complexes and even transcription factors without DNA. Hence statistical analysis of correlations between transcription factors and their sites becomes both a possibility and a necessity.

Transcription factors (TFs) from one structural family tend to recognize similar DNA motifs [8, 28, 29] and that allows one to construct family-specific motifs that may be used both for the identification of candidate binding sites (BS) and for the classification of transcription factors [30]. The correlation between the level of conservation of specific residues in DNA-binding proteins and that of DNA sites has been demonstrated for 21 protein families [31]. The residues contacting the sugar-phosphate backbone are conserved, whereas the residues contacting nucleotide bases are conserved if binding motifs are similar for all proteins from a family, and variable otherwise. Within a genome, there is a correlation between the degree of conservation of a consensus nucleotide and the number of contacts it forms with DNA [32]. In the TAL-effector family of *Xanthomonas* TFs, injected into plant cells during infection, there exists a recognition code linking pairs of amino acid residues, so-called repeat-variable diresidues, and base pairs in the recognized site [33, 34], and this code may be used to predict TAL-effector targets [35, 36]. A similar code was suggested for the Cro family of phage TFs [37].

These and similar observations formed a base for the identification of specificity-determining positions in aligned, homologous protein sequences divided into groups by specificity towards ligands, cofactors or DNA motifs [38]. For each alignment column, the mutual information is calculated as a measure of correlation between the positional amino acid distribution and the division into specificity groups. This method was applied to identification of specificity-determining positions in prokaryotic [38, 39] and eukaryotic [40] transcription factors, and the predictions were in good agreement with the structural and mutagenesis data. The main drawback of the method, the need to define specificity groups in advance, may be partially offset by automated clustering of protein sequences [40, 41].

Similar methods based on measuring the mutual information are widely used for the identification of protein-protein interactions (e.g. [42, 43]) or even prediction of the protein three-dimensional structure [44]. They do not require structural or phylogenetic information. Such methods were applied to identify a fraction of functionally important contacts in several families of eukaryotic TFs [45, 46] and the LacI family of bacterial TFs [28]. A caveat is that this method requires large training samples and an estimate of expected mutual information. It also, by construction, underestimates the importance of conserved positions. One more problem is that it is sensitive to shared evolutionary history of the analyzed factors (phylogenetic trace), and special techniques need to be developed to get rid of the latter [38, 43]. A related approach, applied to the EGR subfamily of eukaryotic zinc finger TFs [47] and to bacterial LacI and TetR families [48], is assigning interaction energies to contacting pairs of residues and bases, and it may suffer from similar drawbacks. Direct analysis of available structures supplemented with calculation of a physical energy function was used to redefine binding motifs for 67 yeast TFs [49, 50]. Binding specificity predictions derived from 3D structures are systemized in the 3D-footprint database [51].

Predicted specific interactions were used to construct mutant TFs with new specificities for a variety of families, both eukaryotic, e.g. zinc fingers [52, 53] and bHLH [54], and prokaryotic, such as TAL effectors [55], LacI [28], and CRP/FNR [56]. On the other hand, extensive experimental screens sometimes produced discouraging results: randomization of DNA-interacting residues of a zinc-finger protein Zif268 [57] and LacI-family TFs [58] did not yield consistent, family-specific protein-DNA interaction codes. Most residues, including non-contacting ones, were shown to influence binding of LacI-family TFs [59, 60]. Contacting residues are not sufficient to explain binding specificity of eukaryotic FOX (forkhead box) TFs [61].

Previously we adapted a number of techniques used to identify specificity determining positions [39] to the identification of correlated protein and nucleotide positions, likely important for protein-DNA recognition. In addition to simple computation of mutual information, our algorithm assesses statistical significance correcting for (possible) overrepresentation of closely related TFs and common ancestry (phylogenetic trace) of some subgroups in a dataset. An objective threshold is set based on probabilistic calculations (the so-called Bernoulli threshold). The algorithm was implemented as a web server Prot-DNA-Korr (http://bioinf.fbb.msu.ru/Prot-DNA-Korr) and applied to study co-evolution of TFs and binding motifs in the NrtR [62] and Rex [63] families of TFs. Here we describe it in detail and apply to the MerR family of bacterial TFs.

### MerR family

TFs from the MerR family regulate response to various stresses: antibiotics, heavy metals, oxidative stress [64], nitrosative stress [65, 66], heat shock [67, 68], carbonyl stress [66, 69, 70], as well as polyamine degradation [71], nitrogen metabolism [72], carotenoid biosynthesis [73], curli and biofilm formation [74], degradation of isoprenoids [75] and branched-chain amino acids [76]. In particular, the family contains a group of TFs that act as transcriptional activators of heavy metal resistance (HMR) systems. These HMR regulators form a distinct cluster within the MerR family (GenBank CDD accession number cl02600). The spectrum of toxic metals includes mercury, copper, zinc, cadmium, lead, silver, and gold.

Experimentally studied proteins, MerR, HmrR, CueR, ZntR, CadR, PbrR, GolS use mono- and divalent metal ions as ligands [64, 77, 78]. In addition, several heavy-metal resistance regulons (sets of operons regulated by particular TFs) were subject for a comparative-genomics study [79]. The binding sites of these TFs are located between the promoter −35 and −10 boxes of the regulated operons, an arrangement being typical for MerR-family transcriptional activators. Moreover, the distance between the promoter boxes in such promoters equals 19–20 bp instead of usual 16–17 bp [64, 69, 70, 79, 80]. The mechanism of transcriptional activation is known from structural and mutational studies [81]. DNA untwisting and base pair distortion decrease the distance between the promoter boxes and set them in a conformation capable of binding by the RNA polymerase. This distance change approximately equals 2 bp. Deletion of 2 bp from the promoter spacer has the same effect on transcription.

The crystal structures in complexes with DNA are known for six MerR-family proteins: BmrR [81–84], MtaN [82] and GlnR [85] from *Bacillus subtilis*, TnrA from *Bacillus megaterium* [85], SoxR from *Escherichia coli* [86, 87], and TipAL from *Streptomyces lividans* (PDB ID 2VZ4). None of them are involved in heavy metal resistance. DNA-free structures are available for BmrR [88] and Mta [89] from *B. subtilis*, CueR and ZntR from *E. coli* [90], NmlR from *Bacillus thuringiensis* (PDB ID 3GPV), BC_0953 *from Bacillus cereus* (PDB ID 3HH0), LMOf2365_2715 (PDB ID 3GP4) and lmo0526 (PDB ID 3QAO) from *Listeria monocytogenes*, and SCO5550 from *Streptomyces coelicolor* [91]. These structures show that TFs from the MerR family have very similar spatial conformations, GlnR, TnrA and SCO5550 being exceptions. The DNA-binding winged helix-turn-helix (WHTH) domain is located in the N-terminus followed by the antiparallel coiled coil providing dimerization. The ligand-binding domains located in the C-terminus may differ in length, sequence and structure. SCO5550 has a different dimerization domain resulting in a different overall structure. GlnR and TnrA have a dimerization domain located in the N-terminus that results in a different mode of interaction between monomers also yielding a different overall dimer architecture. Similar crystal structures and promoter organization suggest that the mechanism of transcriptional activation is the same for all MerR-family activators sharing this structural organization.

## Methods

Here we describe an outline of the algorithm for the identification of correlated pairs of positions. The details for each step are presented in the Results section. The program takes TFs and TFBSs alignments as an input. For each pair of alignment positions we calculate the frequencies of ‘nucleotide—amino acid’ (NT-AA) pairs. From the observed and expected (under hypothesis of independence) frequences we derive a measure of correlation between pair of columns, *mutual information*. Applying the above steps for randomly generated pairs of columns, we obtain the expected mutual information values, which are then corrected by linear transformation to take into account shared ancestry of sequences as described in [38]. From the observed and expected mutual information values, a measure of statistical significance, Z-score, is then derived. Pairs with top Z-scores are designated as statistically significantly correlated. The actual number of pairs is determined by the Bernoulli cutoff procedure [39].

### Study of MerR-family regulators of heavy-metal resistance

Genomic and protein sequences were taken from GenBank RefSeq database (release 55) [92]. Three-dimensional structures of proteins were taken from the PDB database [93]. The GenBank CDD database [94] was used for classification of transcription factor (TF) into subfamilies. Protein-DNA molecular contacts were taken from the NPIDB database [95]. Van der Waals contacts were taken from the articles in which the structures were published. In the NrtR, Rex, MerR cross-family study, Van der Waals contacts were obtained using the HBPLUS utility [96]. Structure-based multiple protein sequence alignments were built using the PROMALS3D program [97]. Phylogenetic trees were constructed using the MEGA5 package [98]. The GenomeExplorer package [99] was used to build positional weighted matrices (PWMs) and to search genomic sequences for transcription factor binding sites (TFBSs) and promoters. TFBS and operon data were submitted to the RegPrecise database [27]. Ancestral protein and DNA sequences were reconstructed using the PAML package [100]. Sequence logos were generated using the WebLogo program [101].

## Results

### Algorithm for the identification of correlated pairs of positions

The correlation between the residues *A* in an amino acid alignment column *i* and the bases *N* in a nucleotide alignment column *j* is measured using the mutual information:

$$\begin{array}{c} I_{i,j}=\sum_{a\epsilon A}\sum_{n\epsilon N}f_{i,j}\left(a,n\right)log\frac{f_{i,j}\left(a,n\right)}{f_{i,j}^{\text{exp}}\left(a,n\right)} \end{array}$$ (1)

where *f*_*i*,*j*_(*a*, *n*) is the observed weighted frequency of a pair (amino acid *a* in the TF alignment column *i*, nucleotide *n* in the site alignment column *j*) and $f_{i,j}^{\text{exp}}\left(a,n\right)=f_{i}\left(a\right)\times f_{j}\left(n\right)$ is the expected weighted frequency of this pair computed as a product of *f*_*i*_(*a*), the weighted frequency of the amino acid *a* at the column *i*, and *f*_*j*_(*n*), the weighted frequency of the nucleotide *n* at the column *j*.

To estimate the statistical significance of the observed mutual information values, one needs the distribution of mutual information for a random pair of columns $I_{i,j}^{∼}$. In order to obtain it, TF-site pairs are randomly reconnected 10,000 times. Further, a linear transformation is applied to take into account shared ancestries (the phylogenetic trace), as described in [38]. Finally the Z-score, a measure of statistical significance, is calculated as:

$$\begin{array}{c} Z_{i,j}=\frac{I_{i,j}-E\left(I_{i,j}^{∼}\right)}{\sigma (I_{i,j}^{∼})} \end{array}$$ (2)

where $E(I_{i,j}^{∼})$ and $\sigma (I_{i,j}^{∼})$ are the mean and the standard deviation, respectively.

The pairs are ranked by calculated Z-scores, and the top *k* pairs are selected, where *k* is determined by the Bernoulli cutoff procedure [39]. In a nutshell it minimizes the probability (reported as p-value) to observe *k* given Z-scores from the Gaussian distribution.

#### Weighting

To avoid overrepresentation of similar, and closely related sequences, we introduce weights of pairs TF–site as products of weights of individual TF and site sequences: *w*(*rs*) = *w*(*r*) × *w*(*s*).

Here, the number of pairs residue *a*—nucleotide *n* (further denoted by [*a* − *n*]) in column [*i*, *j*] is calculated as the sum of weights of TF–site pairs:

$$\begin{array}{c} N_{i,j}\left(a,n\right)=\sum_{rs\epsilon RS_{i,j}^{a,n}}w\left(rs\right) \end{array}$$ (3)

where $RS_{i,j}^{a,n}$ is the set of TF–site pairs with the pair [*a* − *n*] in the columns [*i*, *j*].

Similarly, residues *a* in the column *i* are counted as:

$$\begin{array}{c} N_{i}\left(a\right)=\sum_{rs\epsilon RS_{i}^{a}}w_{rs} \end{array}$$ (4)

where $RS_{i}^{a}$ is the set of TF–site pairs with the residue *a* at position *i* in TF.

Weights of TFs are determined using the Gerstein-Sonnhammer-Chothia algorithm [102]. To do that, the phylogenetic tree of TFs was constructed using the neighbor-joining method implemented in Clustal [103] and rooted in the middle of the longest path between leaves.

#### Pseudocounts

To account for non-observed data and to avoid null frequencies, we introduced pseudocounts supplementing the set of *N* observed sequences by $\kappa \sqrt{N}$ random sequences with amino acid and nucleotide frequencies drawn from the respective alignment columns. At that, the amino acid pseudocounts reflected the amino acid substitution matrix, as in the SDPPred algorithm [39], and the normalized frequency of the amino acid *a* in the alignment column *i* was defined as:

$$\begin{array}{c} f_{i}\left(a\right)=\frac{N_{i}\left(a\right)+\frac{\kappa }{\sqrt{N}}\sum_{b\epsilon A}N_{i}\left(b\right)P\left(b\to a\right)}{N+\kappa \sqrt{N}} \end{array}$$ (5)

where *N*_*i*_(*a*) is the weighted count of amino acid *a* in column *i*, *N* is the total number of residues in the alignment column, *P*(*b* → *a*) is the probability of substitution *b* → *a* computed by the BLOSUM [104] matrix at identity level 30–40%, *κ* = 0.5 is a parameter regulating the contribution of pseudocounts.

The nucleotide pseudocounts are introduced in the same way with substitution probabilities *P*(*m* → *n*) = 1/4 for each pair *m*, *n*.

Finally, the frequency of a pair [*a* − *n*] in columns [*i*, *j*] is computed as:

$$\begin{array}{c} f_{i,j}\left(a,n\right)=\frac{N_{i,j}\left(a,n\right)+\frac{\kappa }{\sqrt{N}}\sum_{b\epsilon A}\sum_{m\epsilon N}N_{i,j}\left(b,q\right)P\left(b,m\to a,n\right)}{N+\kappa \sqrt{N}} \end{array}$$ (6)

By our null hypothesis nucleotide and residue substitutions are independent, thus *P*(*b*, *m* → *a*, *n*) = *P*(*b* → *a*) × 1/4 and:

$$\begin{array}{c} f_{i,j}\left(a,n\right)=\frac{N_{i,j}\left(a,n\right)+\frac{\kappa }{4\sqrt{N}}\sum_{b\epsilon A}P\left(b\to a\right)N_{i}\left(b\right)}{N+\kappa \sqrt{N}} \end{array}$$ (7)

#### Implementation

The algorithm is implemented in the Java language and thus can be executed on any computer provided a Java virtual machine is installed. The program and the source code may be accessed from the web at http://bioinf.fbb.msu.ru/Prot-DNA-Korr. Calculated Z-scores are graphically represented via an interactive heatmap plot (TFs vs TFBSs). A detailed NT-AA contingency table for a requested pair of positions can be drawn for in-depth analysis. Under- and overrepresented NT-AA pairs in the table are emphasized by coloring based on an arbitrary *χ*^2^-score summand cutoff (50 by default). The contingency tables, along with the tables of *χ*^2^ and mutual information summands, as well as list of Z-scores may be exported as a plain text.

### Analysis of heavy-metal resistance regulators from the MerR family

#### Identification of transcription factors and construction of motifs

Proteins containing HTH_CueR, HTH_MerR1, HTH_CadR-PbrR, HTH_CadR-PbrR-like and HTH_HMRTR conserved domains (Specific Protein option in GenBank CDD) were downloaded from the GenBank RefSeq database. Further in this study they are referred to as TFs from the CueR, MerR, CadR-PbrR, CadR-PbrR-like and HMRTR subfamilies, respectively. Only proteins encoded in completely assembled genomes were retained for the analysis. In total they contained 1516 TFs (see Table 1 for details). TFs with sequences longer than 190 bp and shorter than 110 bp were excluded from the study, given that typical proteins of these subfamilies have the length of 130–140 bp [79, 90]. Structure-based multiple sequence alignments were constructed using structural information from CueR (PDB ID 1Q05, 1Q06, 1Q07) and ZntR (PDB ID 1Q08, 1Q09, 1Q0A) from *Escherichia coli* [90]. Phylogenetic trees for each subfamily were built by the neighbor-joining method with pairwise gap deletion option that keeps the information from gap-containing columns. BmrR from *Bacillus subtilis* (GI 50812267) was used as an outgroup. Only one of each group of nearly identical proteins (distance between the leaves on the tree less than 0.02) encoded in genomes of different strains of the same species was retained for further study. Following the application of these procedures, 906 TFs remained in the studied set (Table 1). Most of them (783 TFs) are encoded in genomes of Proteobacteria. Other phyla represented in this set include Actinobacteria (52 TFs), Cyanobacteria (22 TFs), and Firmicutes (18 TFs).

**Table 1:** TFs and TFBSs statistics.

Subfamily	TFs (counts)	TFs after filtering	TFs with identified TFBSs	TFBSs
CueR	511	260	238	324
MerR	205	123	105	106
CadR-PbrR	253	193	172	174
CadR-PbrR-like	189	147	100	110
HMRTR	358	183	148	170
Total	1516	906	763	884

We built selective PWMs for searching the genomes for putative TFBSs using sites from [79] as a starting point. One PWM per subfamily was built with the exception for MerR and HMRTR where a single PWM did not provide desired sensitivity. Hence, two PWMs were constructed for the MerR subfamily and three for the HMRTR subfamily, each corresponding to a separate smaller branch on the phylogenetic tree of studied TFs. The PWMs are presented in S1 File. The length of CueR, CadR-PbrR and CadR-PbrR-like motifs was 21 bp, whereas the length of MerR and HMRTR motifs was 22 bp. The selected genomes were searched for TFBSs in regions from −400 to +50 bp relative to the gene translation start sites annotated in GenBank. The threshold for TFBS search was set to 3.5.

#### Exclusion of false positive TFBS

Numerous experimental and computational studies of promoters regulated by transcriptional activators from the MerR family show that these TFs bind specific sites located between the promoter boxes of the regulated operons [64, 69, 70, 79, 80]. Moreover, the distance between the promoter boxes in such promoters equals 19–20 bp instead of usual 16–17 bp. Previous studies [105] demonstrated that the distance between the center of the TFBS and the 3’-end of the −35 promoter box is fixed within a subfamily. Putative promoters were found using the *E. coli* *σ*70 promoter consensus TTGACA-()-TATAAT. The distance between the TFBS centers and the −35 promoter boxes equals 7 bp for CueR, CadR-PbrR and CadR-PbrR-like sites (21-bp long) and 8 bp for MerR and HMRTR sites (22-bp long). TFBSs scoring above the threshold were considered false positives if they did not overlap with candidate promoters having 19–20 bp spacers or the distance between the center of the site and the 3’-end of the −35 promoter box is other than 7 bp for 21-bp sites and 8 bp for 22-bp sites. Further, only sites co-localized with the TF gene and/or located upstream of genes with relevant function (heavy metal resistance) were retained.

Using this procedure, 884 TFBSs were identified for 763 TFs (Table 1, S2 File). We tested how the usage of site and promoter overlap affects the number of found sites. We did this for weak sites (with scores from 3.5 to 5.0) and strong sites (with scores above 5.0). For strong sites, the number of candidates grows only slightly when the promoter information is omitted. In contrast, for weak sites this number grows tremendously. On average, 97% of candidate sites in a genome are weak sites without promoter support (S3 File). Therefore we used the information about putative promoters.

**Fig 1:**
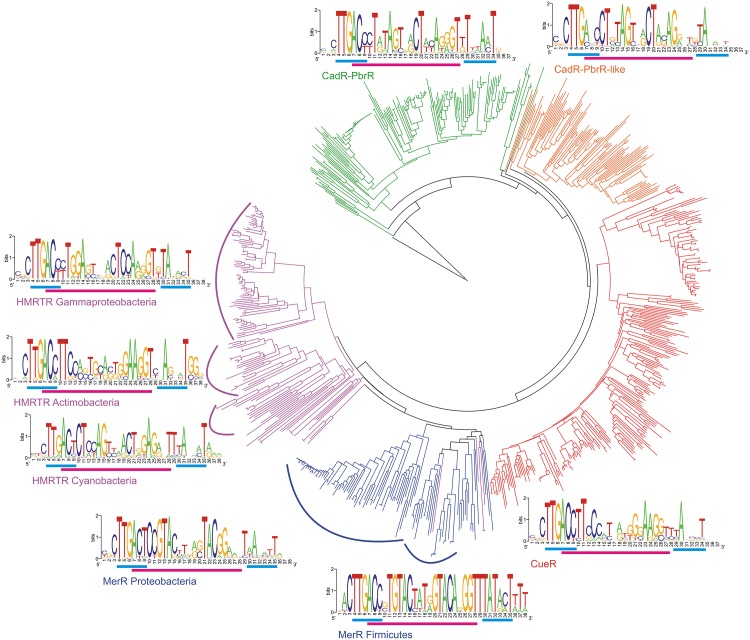
Phylogenetic tree of TFs from studied subfamilies. Subfamily branches are colored: CueR—red, MerR—blue, CadR-PbrR—green, CadR-PbrR-like—orange, HMRTR—purple. Sequence Logos represent binding motifs (magenta bars) with −10 and −35 promoter boxes (cyan bars) and 3 flanking positions.

Sequence Logos for the sites of each studied subfamily are presented in Fig 1. Each Logo includes the binding motif as well as the −35 and −10 promoter boxes and three flanking positions. Then we built the phylogenetic tree for TFs with identified sites (Fig 1). TFs from different subfamilies form distinct branches on the tree with only several exceptions, in agreement with manually curated conserved-domain classification of HMR TFs from the MerR family provided in GenBank CDD (GenBank CDD accession number cl02600). The identified TFBSs were then aligned: one central position was deleted from the 21-bp long sites, and two, from the 22-bp long sites. For the computation of correlations, the alignment block containing 74 columns was taken from the alignment of TFs of all studied subfamilies. This block completely covers the N-terminal DNA-binding winged helix-turn-helix (WHTH) domain of these proteins. A set of corresponding pairs of protein and DNA sequences was formed by this block and the alignment of TFBSs. After deleting duplicate pairs, we obtained a set containing 776 unique pairs of corresponding protein and DNA sequences.

#### Identification and analysis of correlated positions

**Fig 2:**
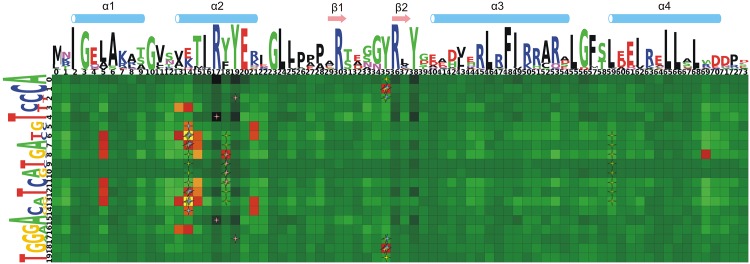
Heatmap of protein-DNA correlations. TF positions are along the horizontal axis and at the Logo above. Site positions are along the vertical axis and at Logo on the left. The color denotes the Z-score for a pair of positions with the color palette for significantly correlated pairs in the yellow to red interval, while black through light green colors denoting positions below the significance threshold. Protein side chain—DNA base interactions are shown as stars: blue—hydrogen bonds; red—Van der Waals contacts; yellow—water bridges; green—hydrophobic contacts. Interactions observed in the structures of complexes at least once are shown. Elements of protein secondary structure (from the crystal structure of *E. coli* CueR—PDB ID 1Q05) are shown at the top.

At the B-cutoff step (S1 Fig), Prot-DNA-Korr suggested 32 correlated pairs corresponding to the global minimum of the p-value. Correlation Z-scores are listed in S4 File. The heatmap showing the correlated positions is presented in Fig 2. This heatmap shows imperfect symmetry due to imperfect symmetry of TF binding sites and respective binding motifs. We searched the literature and the NPIDB database [95] for the contacts of TFs from the MerR family with DNA (Fig 2). All protein-DNA contacts (side chains to bases, side chains to DNA backbone and protein backbone to DNA backbone) are presented in S2 Fig overlaid with the same heatmap. A pair of positions was marked as interacting if the interaction was reported at least once. Since CueR and ZntR structures were resolved in the DNA-free form [90], the experimental contacts come from crystal structures of proteins from subfamilies not included in the present study [81–85, 87]. However, these experimental data are relevant, as the structures of WHTH DNA-binding domains of the TFs from the MerR family are conserved [82, 85, 87, 91]. These crystal structures of dimeric TFs (except MtaN and GlnR from *B. subtilis*) consist of one monomer and one DNA strand. The GlnR structure includes one monomer and one double-stranded half-site and MtaN structure includes both monomers and complete double-stranded site. Therefore we performed a mirror reflection of the contacts to cover both half-sites. This results in a strictly symmetrical map of contacts (Fig 2, S2 Fig).

Overall, 36 experimentally identified interacting pairs (side chain to base) were found. Nine pairs appear as both correlated and forming side-chain-to-base contacts (Fisher’s exact test *p*-value of 1.96 × 10^−8^). This proves the relevance of the applied procedure and cutoff selection. Of 32 correlated pairs, 23 are located in the recognition *α*-helix of the HTH domain of MerR-family proteins (positions 13–21 in the protein alignment in Fig 2). Other correlated pairs correspond to the *α*1-helix of the WHTH domain and the *β*-hairpin between the *β*1 and *β*2 strands that constitutes the first wing of the WHTH domain. The most significantly correlated pairs of positions are symmetrical (6,14) and (13,14). Other correlations are much less significant.

Hereinafter pairs of positions are referred to as (*j*, *i*), where the TFBS position comes before the comma and the TF position, after. Symmetrical TFBS positions give 19 when summed. The ‘nucleotide—amino acid’ pairs for the respective pairs of positions are denoted as NT-AA.

Over- and underrepresented NT-AA pairs along with subfamilies where they preferably occur are listed in Table 2.

**Table 2:** Correlated pairs of positions with over- and underrepresented pairs ‘nucleotide—amino acid’.

Positions	Residues	Count	Type	Note
(6,14)	T-E	200	+	MerR, CadR-PbrR, CadR-PbrR-like
	C-K	191	+	CueR, HMRTR (Actinobacteria)
	G-D	76	+	HMRTR (Gammaproteobacteria)
	A-A	7	+	
	T-K	7	-	
	C-E	3	-	
(13,14)	A-E	184	+	CadR-PbrR, CadR-PbrR-like, HMRTR
	G-K	180	+	CueR, HMRTR (Actinobacteria)
	C-D	79	+	HMRTR (Gammaproteobacteria)
	T-R	9	+	
	A-K	10	-	
	G-E	1	-	
(8,15)	A-M	104	+	CueR
	G-M	3	-	
	A-T	6	-	
(11,15)	T-M	102	+	CueR
	C-M	1	-	
	T-T	4	-	
(3,13)	C-V	208	+	MerR, CadR-PbrR, CadR-PbrR-like
	T-A	146	+	CueR, HMRTR (Gammaproteobacteria)
	G-K	3	+	
	C-A	12	-	
	T-V	9	-	
(16,13)	A-A	136	+	CueR, HMRTR (Gammaproteobacteria)
	A-V	6	-	
(12,15)	G-M	93	+	CueR
	T-M	13	-	
	G-T	13	-	
(7,15)	C-M	86	+	CueR
	A-M	18	-	
	C-T	16	-	
(5,14)	G-E	190	+	MerR, CadR-PbrR, CadR-PbrR-like
	C-K	152	+	CueR, HMRTR (Actinobacteria and Cyanobacteria)
	A-Q	38	+	CadR-PbrR-like
	G-K	24	-	
	C-E	1	-	
(14,14)	C-E	211	+	MerR, CadR-PbrR, CadR-PbrR-like
	G-K	143	+	CueR, HMRTR (Actinobacteria)
	T-Q	27	+	
	T-V	13	+	
	G-E	4	-	
(6,15)	C-M	106	+	CueR
	A-Q	6	+	
(13,15)	G-M	96	+	CueR
(6,21)	T-R	159	+	MerR, CadR-PbrR, CadR-PbrR-like
	C-S	64	+	CueR
	C-E	56	+	CueR
	C-R	7	-	
(13,21)	A-R	149	+	MerR, CadR-PbrR, CadR-PbrR-like
	G-E	53	+	CueR
	C-K	61	+	HMRTR (Gammaproteobacteria)
(7,5)	C-A	87	+	CueR
	C-L	5	-	
(12,5)	G-A	95	+	CueR
	G-L	6	-	
(14,21)	G-R	3	-	
(5,21)	C-R	4	-	
(3,14)	T-K	191	+	CueR, HMRTR (Actinobacteria)
	A-H	2	+	
	C-K	18	-	
	T-E	23	-	
(6,14)	A-K	186	+	CueR, HMRTR (Actinobacteria)
	C-H	2	+	
	G-K	22	-	
	A-E	16	-	
(12,14)	G-K	124	+	CueR, HMRTR (Actinobacteria)
(7,14)	C-K	127	+	CueR, HMRTR (Actinobacteria)
	A-K	67	-	
(8,5)	A-A	97	+	CueR
	A-L	6	-	
(11,5)	T-A	93	+	CueR
	T-L	6	-	
(13,5)	G-A	110	+	CueR
	G-L	35	-	
(6,5)	C-A	117	+	CueR
(1,35)	A-Q	26	+	
	T-R	22	+	
(18,35)	T-H	11	+	
	A-I	17	+	
	A-R	15	+	
(6,13)	C-A	123	+	CueR
(13,13)	A-V	163	+	MerR, CadR-PbrR, CadR-PbrR-like, HMRTR (Cyanobacteria)
	G-A	108	+	
(8,18)	A-H	47	+	
(8,69)	A-W	106	+	CueR

Pairs of positions are ordered by decrease of statistical significance. ‘Residues’ column shows pairs of residues. In ‘Type’ column ‘+’ stands for overrepresented pair, ‘-’ stands for underrepresented pair. ‘Notes’ column shows preferred occurrence of the ‘nucleotide—amino acid’ pair.

We mapped correlated pairs on the phylogenetic tree of the studied TFs (Fig 1), using only pairs where several overrepresented pairs of residues had large (over 50) counts: (3, 13)—S3 Fig, (5,14)—S4 Fig, (6,14)—S5 Fig and (6,21)—S6 Fig. These data show that the same overrepresented pairs NT-AA appeared several times independently in course of evolution. We tested whether mutations in the TF DNA-binding domains lead to subsequent changes in binding motifs. At that, we reconstructed ancestral sequences of studied TFs and their binding sites in internal nodes of the phylogenetic tree of the TFs (data not shown). We used the Jones-Taylor-Thornton (JTT) substitution model for amino acids and general time-reversible (REV/GTR) model for nucleotides. However, we could not observe a prevalence of either protein–DNA or DNA–protein order of mutations leading to the formation of overrepresented pairs.

### Algorithm performance analysis

#### Input data bootstraping

We studied to what extent our method tolerates inadequate data in the input. For that, we progressively shuffled residues in 10%, 20%, etc. of aligned protein sequences, simulating misalignment and wrong input data. Each progressive step was performed 100 times independently. For each pair we calculated the number of its occurrences in the top 32 correlated pairs, which corresponds to the previously established significance threshold.

Bootstrap Table 3 shows that half of 32 significantly correlated pairs remain in the list even if 50% of the data is scrambled. Moreover, top two correlated pairs remain in the list with only 30% of the valid input data. On the other hand, the weaker 1/3 of the list fall below the threshold with only 10% of scrambled data. While the ranks of the said pairs usually drop only slightly below the 32 rank threshold (S5 File), this happens in a consistent manner. For instance, the (18,35) pair originally having rank 31 never gets to the top 32 pairs with 10% of the data scrambled.

**Table 3:** Occurrence of the pair in the top 32 pairs of the list with fraction of the input being scrambled over 100 iterations.

pair	0	0.1	0.2	0.3	0.4	0.5	0.6	0.7	0.8	0.9	1
(6,14)	100	100	100	100	100	100	99	90	46	12	3
(13,14)	100	100	100	100	100	100	98	85	40	5	2
(3,13)	100	100	100	100	100	99	87	67	35	14	5
(11,15)	100	100	100	100	99	100	91	88	63	37	7
(8,15)	100	100	100	100	98	97	90	81	52	29	3
(5,14)	100	100	100	99	98	86	60	40	18	5	1
(16,13)	100	100	100	97	87	83	53	45	13	12	2
(3,14)	100	100	99	99	95	89	72	55	26	6	7
(8,69)	100	100	99	96	94	95	85	72	56	31	10
(16,14)	100	100	98	98	93	82	70	37	27	7	2
(14,14)	100	100	97	94	83	71	43	25	7	2	1
(12,15)	100	99	100	100	98	98	93	81	59	28	10
(7,15)	100	99	100	99	97	94	82	66	46	14	2
(12,14)	100	98	91	86	74	73	41	36	14	8	4
(7,14)	100	96	90	87	62	62	39	29	19	10	2
(6,21)	100	95	74	68	52	42	32	15	11	7	5
(13,21)	100	87	67	50	41	26	29	10	9	4	3
(14,21)	100	87	56	38	20	21	11	9	2	1	0
(5,21)	100	84	61	41	31	24	12	6	3	1	1
(6,15)	100	73	78	77	75	67	59	33	27	7	2
(6,13)	100	55	57	40	33	15	20	16	13	6	0
(13,13)	100	49	34	33	29	13	15	12	9	6	1
(12,5)	100	48	36	28	40	29	18	19	13	5	0
(7,5)	100	41	39	26	31	24	17	18	13	1	3
(1,35)	100	35	33	24	19	12	5	2	2	0	1
(13,15)	100	32	39	45	44	40	28	28	20	6	4
(8,5)	100	11	17	24	29	27	24	23	16	10	3
(11,5)	100	6	12	11	26	24	12	24	22	11	3
(13,5)	100	3	4	9	18	12	13	14	12	5	5
(8,18)	100	2	11	12	11	22	18	10	18	14	2
(18,35)	100	0	3	3	2	4	5	6	2	1	0
(6,5)	99	1	4	8	12	17	12	12	8	4	2
(11,69)	1	100	100	92	96	91	84	70	63	38	5
(12,69)	0	88	80	74	75	55	53	44	30	18	5
(6,69)	0	78	66	45	52	33	41	23	17	10	4
(11,12)	0	72	60	56	45	41	47	20	28	11	5
(13,1)	0	64	79	70	61	49	32	30	7	7	1
(6,1)	0	60	72	72	51	56	36	27	20	4	5
(7,1)	0	54	49	48	44	40	32	28	15	12	3
(7,69)	0	53	47	37	20	23	20	19	12	10	5
(13,69)	0	52	37	26	31	17	23	13	14	10	4

The bootstrap table suggests that the bottom 1/3 of the correlated list are sensitive to the input data quality and, together with some pairs falling just below the significance threshold may be considered as the “grey area”.

We also performed negative control of our method by providing shuffled regulator-site pairs from the initial input data. The shuffling was performed similarly to shuffling for expected mutual information required for Z-scores calculation. The B-cutoff global minimum *log*(*p* − *value*) = −14 (S7 Fig) obtained is negligible compared to −1250 yielded by the original data (S1 Fig).

#### Conservation and correlation

**Fig 3:**
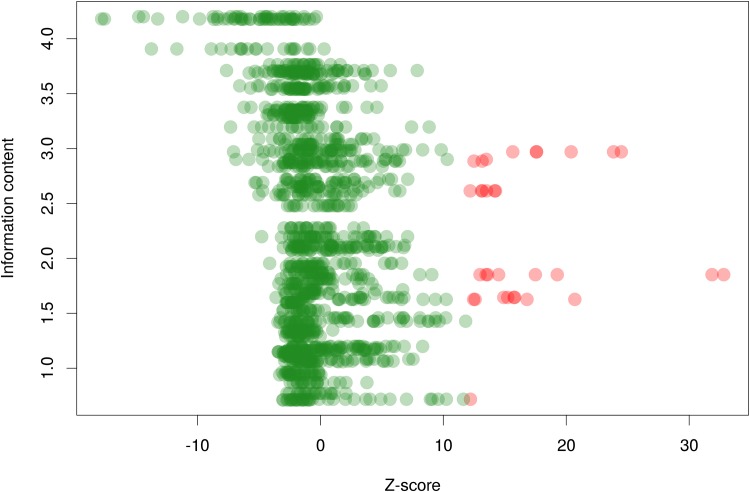
Z-score vs. protein column conservation. Red—significantly correlated pairs. Green—other pairs. Y-axis is the protein positional information content for corresponding pair of columns after weighting and adding pseudocounts. X-axis is the Z-score of a pair.

Protein positional information content used in the logo generation as a measure of conservation was compared with Z-scores for corresponding pairs of columns. The correlated protein positions appear to be moderately conserved. Fig 3 suggests that overall highly conserved residues tend to have lower z-scores.

Conserved correlated residues that form contacts are very rare. In three PDB (3ikt, 3gz6, 1r8e) structures from the Rex, NrtR and MerR families, respectively, we found 43 contacts with either of the partners being conserved (S6 File). Only two such contacts in the NrtR structure appeared to be correlated.

## Discussion

We developed and implemented an algorithm for the identification of pairs of positions that are important for the protein-DNA recognition. Our method requires multiple alignments of DNA-binding proteins and of their respective sites. The method does not rely on known 3D structures of protein-DNA complexes, here we rather use them to validate our results. It should be noted that of necessity the contacts and correlations were identified on different sets of TFs belonging to different subfamilies.

The comparison with structural data shows good agreement both in quantitative and qualitative terms. The sets of correlated and contacting pairs strongly overlap (Fisher’s test p-value 1.96 × 10^−8^). The recognition helix of the HTH domain contains a large cluster of correlated pairs. According to classic Suzuki studies of spatial structures [2], residues 1, 2, 6 of the recognition helix that face the DNA major groove are most important for the protein-DNA recognition as they form hydrogen bonds with DNA bases hence allowing the protein to read the DNA sequence. Here these residues participated in correlated pairs, with residue 2 being the most correlated. The MerR and previously studied Rex [63] and NrtR [62] families provide correlation data on three families HTH binding domains. Residue 6 participates in correlations in all three families and residues 1, 2 in two familes each.

Among hydrogen bonds, Van der Waals interactions, and hydrophobic contacts in the three families we do not see any preference for either type to correspond to correlations (S5 File). However, the data are not sufficient to form a solid conclusion.

Although significantly correlated pairs are likely to be contacting ones, our algorithm is not merely a substitute for a 3D structures analysis. Conserved interactions will not demonstrate correlations due to the lack of sequence variation [45]. On the other hand, some residues may affect specificity indirectly, and it would be difficult to identify them in 3D structures. The correlation analysis identifies all coevolving pairs of positions and along with overrepresented NT-AA pairs thus providing hints for future experimental investigations [56].

In most correlated pairs of positions, overrepresented NT-AA pairs appear independently multiple times in course of evolution of the studied TFs. It has been shown that binding sites for existing TFs can emerge rather rapidly from sequences that resemble weak sites [106, 107]. This model implies that changes in a TF sequence, decreasing its affinity to pre-existing sites, yield changes in the sites, hence restoring the effective binding. The binding motif (in the simplest form, the consensus of the sites) changes accordingly. We reconstructed ancestral sequences of TFs and the respective DNA motifs, but failed to confirm the hypothesis about the leading role of substitutions in TFs yielding subsequent substitutions in recognized sites and hence motifs.

We used several crystal structures of related TFs in the DNA-bound form to demonstrate high level of coincidence between correlated pairs and contacting positions. At that, it is plausible that the conserved positions provide for the initial DNA binding, whereas correlated positions fine-tune interactions with specific sites. A proof of concept was provided by an experimental study of CRP, that demonstrated lack of specific binding after individual mutations in either the TF or the site, but partially reconstituted binding after dual TF-site mutations substituting one preferred NT-AA pair to another pair preferred at the given positions [56]. While existing computational methods may not predict DNA motif given only TF sequence and 3D structure, some progress has been already made. For example, it is possible to match each TF from a given family, present in a genome, to the respective motif from a given set of motifs recognized by these TFs in the same genome [108]. The latter situation arises in comparative-genomic prediction of transcriptional networks.

TFBS prediction and regulon reconstruction in multiple related genomes using comparative genomic approaches has become a major source of information about regulatory networks. Combined with identification of correlations between the sequences of TFs and their binding sites, they may become powerful tools for studying the evolution of TF families and coevolution of interacting protein and DNA sequences using sequence data alone.

## Supporting Information

S1 File Positional weighted matrices (PWMs) used to search the genomes for binding sites. (PDF)

S2 File TF and TFBS data. Data on different subfamilies are presented on separate sheets. Only the first members of regulated operons are shown. TFBS positions are given relative to translation starts of regulated genes annotated in the genomes.
(XLS)

S3 File Distribution of site persentages. Horizontal axis shows the percentage of sites from a given category from all sites found in genome. The vertical axis shows the number of such genomes.
(PDF)

S4 File List of Z-scores for pairs of positions. (PDF)

S5 File Average ranks of pairs after the input data bootstrap procedure. (XLS)

S6 File Contacts correlations and conservation in the Rex, NrtR, MerR families members. (XLS)

S1 Fig B-cutoff plot. Global minimum p-value corresponds to 32 pairs.
(PDF)

S2 Fig Heatmap of protein-DNA correlations with complete map of contacts. TF positions are along the horizontal axis and at the Logo above. Site positions are along the vertical axis and at Logo on the left. The color denotes the Z-score for a pair of positions with the color palette for significantly correlated pairs in the yellow to red interval, while black through light green colors denote positions below the significance threshold. Protein-DNA interactions are shown as stars. Interactions observed in the structures of complexes at least once are shown. Elements of protein secondary structure (from the crystal structure of *E. coli* CueR – PDB ID 1Q05) are shown at the top.
(PDF)

S3 Fig Phylogenetic tree of the TFs from the MerR family with pairs of residues in positions (3,13). Colors of branches show overrepresented pairs of residues in positions (3,13) (see color codein the picture). Background colors show TF subfamilies: red—CueR, blue – MerR, green—CadR-PbrR, beige—CadR-PbrR-like, pink—HMRTR.
(PDF)

S4 Fig Phylogenetic tree of the TFs from the MerR family with pairs of residues in positions (5,14). Colors of branches show overrepresented pairs of residues in positions (5,14) (see color code in the picture). Background colors show TF subfamilies: red—CueR, blue – MerR, green—CadR-PbrR, beige—CadR-PbrR-like, pink—HMRTR.
(PDF)

S5 Fig Phylogenetic tree of the TFs from the MerR family with pairs of residues in positions (6,14). Colors of branches show overrepresented pairs of residues in positions (6,14) (see color code in the picture). Background colors show TF subfamilies: red—CueR, blue – MerR, green—CadR-PbrR, beige—CadR-PbrR-like, pink—HMRTR.
(PDF)

S6 Fig Phylogenetic tree of the TFs from the MerR family with pairs of residues in positions (6,21). Colors of branches show overrepresented pairs of residues in positions (6,21) (see color code in the picture). Background colors show TF subfamilies: red—CueR, blue – MerR, green—CadR-PbrR, beige—CadR-PbrR-like, pink—HMRTR.
(PDF)

S7 Fig B-cutoff plot for shuffled regulator-site pairs. (PDF)
