# Supplementary material for: Identification of Position-Specific Correlations between DNA-Binding Domains and Their Binding Sites. Application to the MerR Family of Transcription Factors
Source: PLoS One. 2016 Sep 30;11(9):e0162681. doi: 10.1371/journal.pone.0162681 (PMC5045206; doi:10.1371/journal.pone.0162681)
Supplement: S1 File — (PDF) [file pone.0162681.s001.pdf]

Supporting material S1. Positional weighted matrices (PWMs) used to search the genomes for binding sites

CueR

```
   a     c     g     t
  0.46  -0.15  -0.15  -0.15
 -0.21   0.40   0.02  -0.21
 -0.23   0.38  -0.07  -0.07
 -0.21   0.02  -0.21   0.40
 -0.15  -0.15  -0.15   0.46
  0.08   0.22   0.03  -0.33
  0.07   0.32  -0.27  -0.12
  0.15   0.34  -0.24  -0.24
  0.10   0.08  -0.06  -0.12
 -0.30   0.11  -0.08   0.27
 -0.03  -0.08   0.04   0.07
  0.23  -0.14   0.21  -0.30
 -0.17   0.04   0.01   0.12
 -0.14  -0.29   0.26   0.17
 -0.06  -0.28   0.31   0.03
 -0.32   0.12   0.24  -0.04
  0.46  -0.15  -0.15  -0.15
  0.36  -0.23   0.10  -0.23
 -0.23  -0.07   0.38  -0.07
 -0.25   0.18   0.32  -0.25
  0.01  -0.10  -0.17   0.26
=MinScore=5.1, MaxScore=6.1
*************************
====== Traning Set: =======
         01   6.12 accttccccttgctggaaggt
         29   6.06 accttcccacagtgggaaggt
         24   6.02 accttgccatggcgggaaggt
         36   6.00 accttcccattattggaagga
         26   5.98 accttcccgttgctggaaggt
         17   5.91 accttaccatgatggcaagct
         15   5.87 accttacccttgagggaaggt
         07   5.86 accttcaccttgctggaaggt
         37   5.85 accttaccattgttggaagga
         30   5.83 accttccaatggtggtaaggt
         05   5.82 accttcccacaatggcaagct
         23   5.78 accttaccctcatgggaagga
         03   5.73 accttaaccttgctggaaggt
         02   5.71 accttcccgtaagggaagga
         21   5.71 accttcccatgatgggaatct
         34   5.69 accttcccatcatgggaagcc
         04   5.69 accttcccgttagggcagggt
         11   5.67 agcttccccttaggggaagct
         10   5.67 agcttccccttaggggaagct
         08   5.66 accttcccccaggggagggt
         28   5.65 accttccagtcgctggaaggt
```

22	5.53	accttaccatgatgtcaagga
19	5.52	accttcccgctggggcagggt
18	5.46	acgttccccttgatggaaggt
35	5.46	accttgccacgatgggaagcc
38	5.43	accttccaacggttggaaggg
09	5.43	accttcccctaagaggagggt
06	5.42	accttccaacactggcaaggt
32	5.40	accttgtcctgatggcaagct
20	5.40	accttacaatcatgacaaggt
16	5.37	accctccccttgctagaaggt
39	5.37	accttccaacgatgggaacct
12	5.37	accttccattaactgtaaggt
27	5.36	accttccatgggctggaaggt
31	5.31	accttgcctgcgtggcaaggt
33	5.30	accttgccatgatgttagggt
13	5.28	actttaaccttgctggaaggt
14	5.11	accttaaccctaggtcaagct
25	5.09	accctgacatgatgtcaaggt

MerR

Proteobacteria

a	c	g	t
0.36	0.00	-0.18	-0.18
-0.20	0.33	-0.20	0.07
-0.18	0.00	-0.18	0.36
-0.14	0.42	-0.14	-0.14
-0.18	0.36	-0.18	0.00
-0.14	-0.14	0.42	-0.14
-0.14	-0.14	-0.14	0.42
0.42	-0.14	-0.14	-0.14
-0.18	0.36	0.00	-0.18
0.05	0.05	-0.28	0.18
0.01	0.01	-0.26	0.24
0.09	-0.13	-0.05	0.09
0.21	-0.22	0.24	-0.22
-0.22	0.18	0.26	-0.22
-0.14	-0.14	-0.14	0.42
0.42	-0.14	-0.14	-0.14
-0.14	0.42	-0.14	-0.14
-0.14	-0.14	0.42	-0.14
-0.18	0.00	0.36	-0.18
0.31	-0.04	-0.04	-0.23
0.18	0.00	0.10	-0.28
-0.06	-0.01	0.04	0.04

=MinScore=5.6, MaxScore=7.0

****

====== Traning Set: =======

06	6.99	actccgtacttaagtacggaag
02	6.88	actccgtacattggtacggaag

```
          01   6.79 actccgtacataactacggaag
          05   6.78 actccgtacttgactacggaag
          08   6.67 attccgtactttggtacggagt
          07   6.65 actccgtaccttggtacggacc
          13   6.48 actccgtacatcggtacggaga
          12   6.44 actccgtactatggtacggcat
          11   6.42 actccgtacttgagtacggggt
          03   6.33 accccgtacctaactacggaaa
          09   6.20 actccgtactcaggtacgcacc
          10   6.18 actctgtaccatgctacggaac
          04   5.63 cttccgtagtcaactacggagt
```

Firmicutes

```
   a      c      g      t
  0.39  -0.13  -0.13  -0.13
 -0.13   0.39  -0.13  -0.13
 -0.18   0.28  -0.18   0.08
  0.03   0.03   0.16  -0.23
 -0.13  -0.13  -0.13   0.39
 -0.13  -0.13   0.39  -0.13
 -0.13  -0.13  -0.13   0.39
  0.39  -0.13  -0.13  -0.13
 -0.13   0.39  -0.13  -0.13
 -0.18   0.08  -0.18   0.28
  0.28  -0.18  -0.18   0.08
 -0.13  -0.13  -0.13   0.39
  0.19  -0.19   0.19  -0.19
 -0.13  -0.13   0.39  -0.13
 -0.13  -0.13  -0.13   0.39
  0.39  -0.13  -0.13  -0.13
 -0.13   0.39  -0.13  -0.13
  0.39  -0.13  -0.13  -0.13
  0.03  -0.23   0.16   0.03
 -0.13  -0.13   0.39  -0.13
 -0.13  -0.13   0.39  -0.13
 -0.13  -0.13  -0.13   0.39
=MinScore=6.7, MaxScore=7.6
***************************
====== Traning Set: =======
          01   7.59 accgtgtactatagtacagggt
          04   7.47 accgtgtactatggtacatggt
          02   7.47 accctgtactatggtacagggt
          03   6.74 actatgtaccttagtacaaggt
```

CadR-PbrR

```
   a      c      g      t
  0.42  -0.14  -0.14  -0.14
```

-0.14	0.42	-0.14	-0.14
-0.22	0.28	-0.22	0.15
-0.07	0.22	-0.26	0.11
-0.14	-0.14	-0.14	0.42
0.23	-0.22	0.21	-0.22
-0.20	-0.20	0.07	0.33
0.42	-0.14	-0.14	-0.14
0.00	-0.18	0.36	-0.18
-0.20	0.07	-0.20	0.33
0.17	-0.26	0.17	-0.08
0.20	-0.26	0.14	-0.08
-0.14	0.42	-0.14	-0.14
-0.14	-0.14	-0.14	0.42
0.24	-0.07	-0.25	0.08
-0.25	0.24	-0.07	0.08
0.36	-0.18	0.00	-0.18
0.12	-0.21	0.30	-0.21
0.12	-0.21	0.30	-0.21
-0.14	-0.14	0.42	-0.14
-0.04	-0.04	-0.22	0.30

=MinScore=5.6, MaxScore=6.7

****

====== Traning Set: =======

07	6.75	accctatagtggctacagggt
06	6.64	accttatagtagctacagggt
16	6.52	accctgtagcaactacagggt
08	6.49	accctatagtaactccagggt
09	6.45	accttgtagtggcttcagggt
02	6.36	actctatagtaactagagggt
11	6.32	actctatagtaactatagagt
05	6.17	actatgtagtgactacaaggt
14	6.00	accttggagtggcttcaaggt
01	5.97	actctatagtgactatagaga
13	5.94	accttatagtagctataaggc
15	5.87	accctgtaatgactacggagt
12	5.57	accctggagcttcttcagggt

CadR-PbrR-like

a	c	g	t
0.24	0.21	-0.23	-0.23
0.17	-0.07	-0.27	0.17
-0.25	0.26	0.04	-0.05
-0.14	0.43	-0.14	-0.14
-0.14	-0.14	-0.14	0.43
0.14	0.30	-0.22	-0.22
-0.21	0.09	-0.21	0.33
0.43	-0.14	-0.14	-0.14
-0.14	-0.14	0.43	-0.14
-0.03	-0.03	-0.23	0.30

```
 -0.11   0.16   0.05  -0.11
  0.27  -0.22   0.18  -0.22
 -0.14   0.43  -0.14  -0.14
 -0.14  -0.14  -0.14   0.43
  0.33  -0.21   0.09  -0.21
 -0.19  -0.19   0.37   0.01
  0.43  -0.14  -0.14  -0.14
 -0.14  -0.14   0.43  -0.14
  0.08   0.01   0.19  -0.28
  0.23  -0.26  -0.06   0.09
  0.11   0.07  -0.29   0.11
=MinScore=4.3, MaxScore=6.5
**************************
====== Traning Set: =======
          02   6.50 aacctctagtcactagagcaa
          07   6.49 ctcctctagtgactagaggac
          06   6.48 ctcctatagtcactagaggaa
          09   6.38 ctcctctagttactagaggat
          05   6.36 aagctctagtcgctagaggaa
          10   6.33 atcctctagtcgctagagatt
          01   6.17 aagctctagtgactagagcaa
          08   6.06 ctcctctagtcgctggagatt
          11   6.04 aacctctagccactagagatc
          04   5.91 aacctacagtagctagaggat
          03   4.31 cctctacagagactgtagggc
```

HMRTR

Gammaproteobacteria

```
   a     c     g     t
  0.40  -0.13  -0.13  -0.13
 -0.13   0.40  -0.13  -0.13
 -0.20   0.23  -0.20   0.18
 -0.20   0.18  -0.20   0.23
 -0.13  -0.13  -0.13   0.40
 -0.01  -0.23   0.25  -0.01
  0.04  -0.18   0.33  -0.18
  0.40  -0.13  -0.13  -0.13
 -0.18   0.04   0.33  -0.18
 -0.18   0.04  -0.18   0.33
  0.08  -0.03  -0.24   0.19
  0.18  -0.20   0.23  -0.20
  0.33  -0.18   0.04  -0.18
 -0.13   0.40  -0.13  -0.13
 -0.13  -0.13  -0.13   0.40
 -0.13   0.40  -0.13  -0.13
 -0.18   0.33  -0.18   0.04
  0.40  -0.13  -0.13  -0.13
  0.28  -0.20   0.12  -0.20
 -0.01  -0.01   0.25  -0.23
```

```
 -0.13  -0.13   0.40  -0.13
 -0.13  -0.13  -0.13   0.40
=MinScore=6.3, MaxScore=7.2
**************************
====== Traning Set: =======
          02   7.18 actctggagttgactccaaggt
          04   6.99 accttgaagttgactccaaggt
          05   6.94 accttggagttaactctaaggt
          07   6.87 accttggagtaggctccaaggt
          01   6.55 actctggagtcgactccagagt
          03   6.43 accttagagctaactccaacgt
          06   6.31 actcttgactaaactccagggt
```

Cyanobacteria

```
   a      c      g      t
  0.40  -0.13  -0.13  -0.13
 -0.13   0.40  -0.13  -0.13
 -0.02  -0.02  -0.23   0.26
 -0.18   0.33  -0.18   0.03
 -0.13  -0.13  -0.13   0.40
  0.05   0.16   0.05  -0.26
 -0.21   0.21  -0.21   0.21
  0.40  -0.13  -0.13  -0.13
 -0.13  -0.13   0.40  -0.13
 -0.13  -0.13  -0.13   0.40
 -0.20   0.11  -0.20   0.29
  0.21  -0.03  -0.24   0.06
  0.25  -0.21   0.16  -0.21
 -0.13   0.40  -0.13  -0.13
 -0.13  -0.13  -0.13   0.40
  0.21  -0.21   0.21  -0.21
 -0.23  -0.02   0.26  -0.02
  0.40  -0.13  -0.13  -0.13
 -0.13  -0.13   0.40  -0.13
  0.29  -0.20   0.11  -0.20
 -0.03   0.06   0.21  -0.24
 -0.13  -0.13  -0.13   0.40
=MinScore=5.9, MaxScore=7.1
**************************
====== Traning Set: =======
          02   7.13 actctctagttaactggagagt
          08   7.05 actctctagttagctggagagt
          03   7.05 actctctagttagctggagagt
          06   6.69 actctacagtctactagagagt
          04   6.69 actctgcagtcaactagagact
          05   6.44 actctacagtttactacagact
          07   6.18 acactccagttcactggaggat
          01   5.89 accttgtagttagctatagggt
```

Actinobacteria

a	c	g	t
0.41	-0.14	-0.14	-0.14
-0.14	0.41	-0.14	-0.14
0.12	0.28	-0.20	-0.20
-0.14	-0.14	-0.14	0.41
-0.14	-0.14	-0.14	0.41
-0.18	0.33	0.04	-0.18
-0.03	0.20	-0.25	0.08
0.28	0.12	-0.20	-0.20
-0.20	0.12	0.28	-0.20
-0.20	0.12	-0.20	0.28
-0.18	0.04	0.33	-0.18
-0.20	0.28	-0.20	0.12
0.28	-0.20	0.12	-0.20
-0.03	0.20	0.08	-0.25
-0.01	-0.23	-0.01	0.25
-0.03	-0.25	0.20	0.08
-0.18	0.04	0.33	-0.18
0.41	-0.14	-0.14	-0.14
0.41	-0.14	-0.14	-0.14
-0.18	0.04	0.33	-0.18
-0.14	-0.14	0.41	-0.14
-0.14	-0.14	-0.14	0.41

=MinScore=5.8, MaxScore=6.8

****

====== Traning Set: =======

01	6.82	accttcccctgcactggaaggt
07	6.79	accttctagtgcactagaaggt
06	6.59	acattccagtgcgatggaaggt
03	6.44	acattccagtgtaggggaaggt
05	6.36	accttcccccgcactggaacgt
02	6.18	accttcaagcctacttgaaggt
04	5.77	accttgtagtgcggatcaaggt
