# Supplementary material for: Identification of Position-Specific Correlations between DNA-Binding Domains and Their Binding Sites. Application to the MerR Family of Transcription Factors
Source: PLoS One. 2016 Sep 30;11(9):e0162681. doi: 10.1371/journal.pone.0162681 (PMC5045206; doi:10.1371/journal.pone.0162681)

Supporting material S3. Distribution of site persentages.

Horizontal axis shows percentage of sites from a given category from all sites found in the genome. Vertical axis shows number of such genomes.

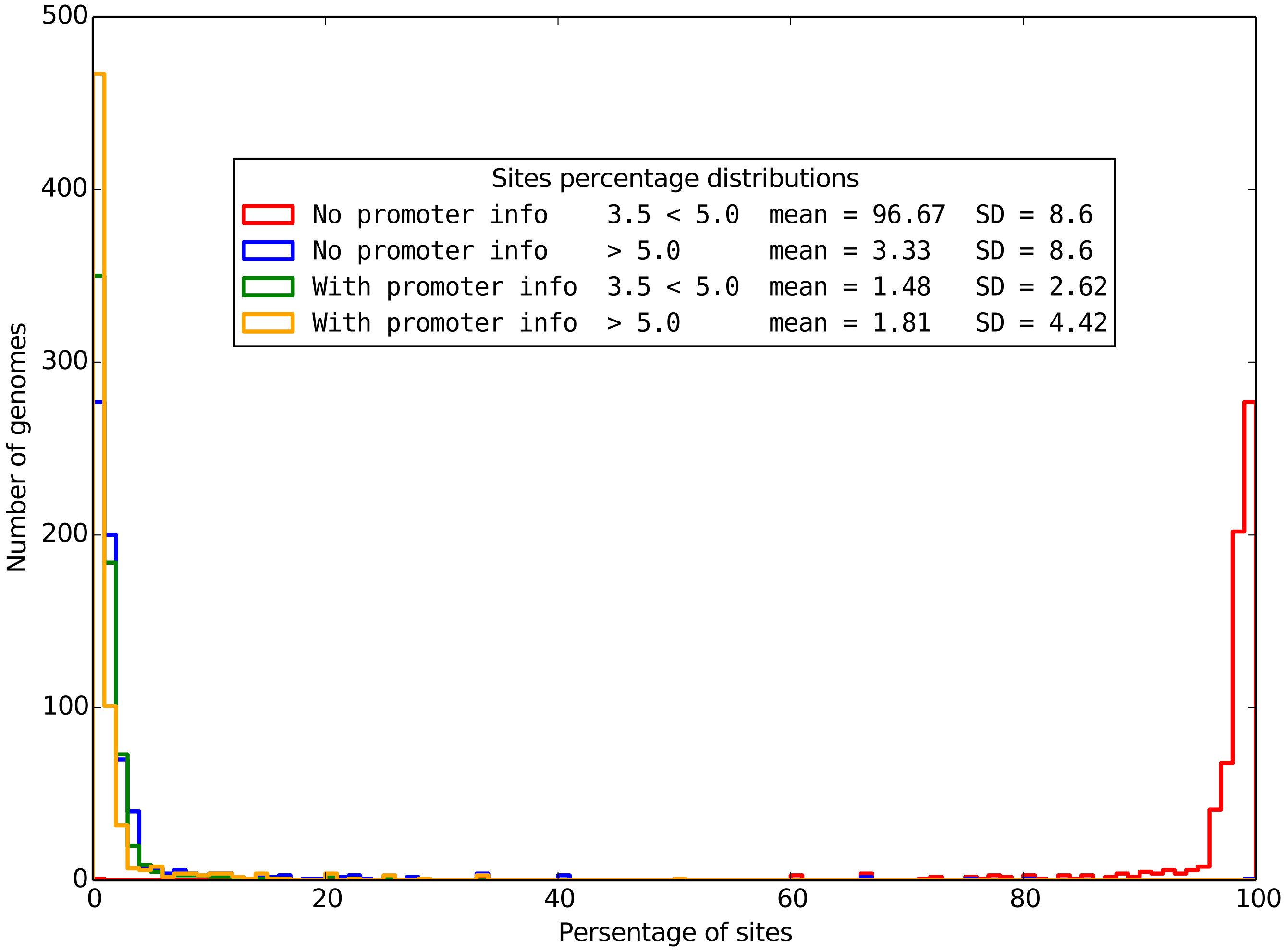

Supplement: S3 File — Horizontal axis shows the percentage of sites from a given category from all sites found in genome. The vertical axis shows the number of such genomes. (PDF) [file pone.0162681.s003.pdf]
