# Supplementary material for: Identification of Position-Specific Correlations between DNA-Binding Domains and Their Binding Sites. Application to the MerR Family of Transcription Factors
Source: PLoS One. 2016 Sep 30;11(9):e0162681. doi: 10.1371/journal.pone.0162681 (PMC5045206; doi:10.1371/journal.pone.0162681)
Supplement: S4 File — (PDF) [file pone.0162681.s004.pdf]

Supporting material S4. List of Z-scores for pairs of positions.

Full correlated list. Created: 13.10.2014
Columns: Nucleotides column index, amino acids column index, Z-score, mutual information, expected mutual information, sigma of mutual information

6	14	31.02	1.103033	0.128167	0.031423
13	14	30.09	1.061747	0.129968	0.030966
8	15	22.50	0.546506	0.070779	0.021141
11	15	21.92	0.551275	0.075133	0.021721
3	13	20.30	0.611765	0.101550	0.025136
5	14	18.56	0.686609	0.118735	0.030590
12	15	18.87	0.457632	0.106036	0.018629
6	15	16.29	0.427129	0.068825	0.021989
7	15	16.06	0.439740	0.105431	0.020813
16	13	16.62	0.565757	0.109679	0.027436
14	14	16.36	0.585798	0.114359	0.028811
6	21	14.95	0.520926	0.105781	0.027778
13	15	14.69	0.380233	0.063092	0.021591
13	21	14.74	0.507710	0.105615	0.027285
14	21	14.75	0.461903	0.098615	0.024632
7	5	12.74	0.318864	0.080589	0.018696
8	5	13.63	0.332646	0.062759	0.019800
12	5	13.90	0.312851	0.079392	0.016791
3	14	12.83	0.473258	0.103338	0.028834
12	14	13.68	0.432603	0.093986	0.024756
5	21	14.01	0.466253	0.103167	0.025911
13	5	12.57	0.314713	0.060183	0.020245
7	14	13.08	0.449479	0.096881	0.026967
16	14	12.52	0.496821	0.111671	0.030762
1	35	12.58	0.439968	0.106519	0.026507
6	5	11.50	0.299752	0.063098	0.020573
11	5	12.46	0.314878	0.065688	0.020003
6	13	12.06	0.469266	0.117646	0.029145
13	13	12.25	0.469321	0.116455	0.028815
8	18	12.89	0.286435	0.054934	0.017957
18	35	11.78	0.394032	0.090044	0.025811
8	69	11.68	0.574810	0.169712	0.034680
11	69	11.08	0.552907	0.169404	0.034613
7	13	9.98	0.345571	0.097493	0.024853
11	12	10.93	0.424125	0.112155	0.028544
11	18	9.84	0.238040	0.058751	0.018225
6	1	8.78	0.463859	0.166101	0.033903
13	1	9.53	0.486104	0.169011	0.033263
8	12	9.22	0.367147	0.110167	0.027878
13	16	8.75	0.134586	0.017802	0.013353
8	53	9.09	0.241712	0.061359	0.019844
6	69	9.05	0.523693	0.186465	0.037277
12	69	9.84	0.375080	0.114854	0.026457
7	1	7.69	0.344720	0.118323	0.029445
7	12	7.80	0.289789	0.100800	0.024218
6	12	8.78	0.381480	0.118728	0.029931
8	13	8.08	0.334585	0.110150	0.027776
8	14	7.61	0.344421	0.118379	0.029695

11	14	8.51	0.375567	0.118848	0.030183
12	13	9.35	0.301732	0.092925	0.022334
13	12	8.40	0.364763	0.116378	0.029577
12	12	8.49	0.286205	0.096422	0.022356
8	22	7.93	0.367934	0.130799	0.029918
11	53	7.74	0.223687	0.064254	0.020608
7	69	8.72	0.368996	0.123644	0.028132
13	69	8.44	0.498454	0.188280	0.036758
3	1	6.49	0.318150	0.128685	0.029172
6	16	7.27	0.123248	0.023926	0.013664
4	20	7.35	0.240762	0.133231	0.014624
3	5	6.24	0.207959	0.088070	0.019210
12	1	6.27	0.285395	0.114793	0.027222
8	21	6.40	0.266725	0.099848	0.026079
6	53	6.19	0.190109	0.058774	0.021211
13	53	6.49	0.182038	0.054882	0.019596
8	59	6.96	0.236709	0.078517	0.022744
6	70	7.38	0.463624	0.193652	0.036593
3	69	6.66	0.313747	0.122606	0.028698
13	70	7.06	0.452235	0.197370	0.036115
8	1	5.14	0.314757	0.153332	0.031401
6	9	5.00	0.245232	0.106047	0.027841
16	1	5.98	0.335875	0.141637	0.032469
8	9	6.37	0.269170	0.100956	0.026410
6	11	6.59	0.223269	0.070352	0.023197
4	12	6.64	0.227026	0.095622	0.019799
16	5	5.78	0.198697	0.079059	0.020700
11	9	5.65	0.253135	0.103070	0.026575
13	9	5.63	0.258111	0.104084	0.027344
11	13	6.49	0.292599	0.111319	0.027939
6	22	5.44	0.317473	0.142132	0.032258
7	21	5.84	0.227187	0.091592	0.023218
16	15	5.30	0.217521	0.097757	0.022609
13	22	5.90	0.328583	0.141653	0.031679
4	31	6.45	0.248886	0.093828	0.024058
11	22	6.50	0.326560	0.131023	0.030075
15	20	5.42	0.217865	0.124442	0.017238
11	21	5.48	0.246840	0.100655	0.026683
12	21	5.27	0.199530	0.087197	0.021333
3	35	5.91	0.238844	0.102688	0.023044
4	35	5.88	0.229095	0.115067	0.019378
3	33	6.45	0.271534	0.105129	0.025806
2	35	6.15	0.236569	0.097575	0.022607
13	28	5.36	0.322670	0.155233	0.031236
11	33	4.81	0.234097	0.105160	0.026815
16	35	4.91	0.217939	0.097744	0.024471
6	44	4.98	0.333093	0.161085	0.034547
11	44	5.73	0.334138	0.145439	0.032959
16	33	4.72	0.240546	0.104501	0.028799
8	45	4.95	0.296191	0.143030	0.030953
8	44	5.34	0.318747	0.146384	0.032252
7	45	5.87	0.269007	0.098485	0.029072
13	56	4.97	0.109871	0.033327	0.015413
11	59	5.55	0.209350	0.080390	0.023227

13	59	5.40	0.208837	0.079310	0.023988
6	0	3.66	0.235791	0.126441	0.029839
11	1	4.39	0.295981	0.152328	0.032739
13	0	4.41	0.256183	0.125945	0.029539
5	5	3.35	0.138300	0.071528	0.019939
3	11	3.85	0.161268	0.084463	0.019966
5	12	4.39	0.236051	0.111322	0.028400
8	11	4.19	0.158731	0.067913	0.021692
3	15	5.03	0.223190	0.115326	0.021423
11	11	4.28	0.164867	0.071025	0.021949
4	21	5.08	0.183195	0.087180	0.018892
6	18	5.08	0.149099	0.052471	0.019024
13	11	5.22	0.185093	0.067214	0.022574
15	12	6.06	0.243167	0.103764	0.022996
4	22	5.06	0.196338	0.089247	0.021146
16	12	4.07	0.225133	0.112340	0.027707
13	18	5.05	0.141842	0.047753	0.018629
12	18	6.04	0.177872	0.085200	0.015343
6	28	6.65	0.364095	0.149943	0.032207
13	25	4.55	0.122339	0.042249	0.017606
4	9	2.78	0.164958	0.110486	0.019593
2	13	3.54	0.194531	0.104339	0.025476
3	12	4.03	0.205985	0.104767	0.025116
15	30	3.65	0.191715	0.098503	0.025551
14	5	2.59	0.119602	0.070261	0.019034
4	14	3.29	0.165626	0.094359	0.021684
5	13	2.72	0.184625	0.109905	0.027514
7	11	3.69	0.150323	0.079608	0.019183
2	20	3.37	0.144904	0.085781	0.017548
7	18	3.64	0.145394	0.085083	0.016552
3	21	3.09	0.169287	0.097463	0.023268
15	14	2.84	0.173215	0.100152	0.025683
6	25	3.42	0.109312	0.045859	0.018578
4	27	3.42	0.167309	0.087914	0.023216
18	13	3.29	0.217161	0.119215	0.029762
4	30	3.51	0.182059	0.102983	0.022510
8	28	3.60	0.247148	0.137899	0.030329
6	45	3.32	0.265394	0.157745	0.032443
13	57	4.25	0.130826	0.048223	0.019432
8	42	3.19	0.219586	0.124810	0.029712
15	35	3.36	0.191922	0.113985	0.023176
6	49	3.82	0.086096	0.028915	0.014970
13	45	4.18	0.294844	0.161485	0.031929
13	44	4.96	0.332444	0.162259	0.034290
12	45	4.14	0.208662	0.096099	0.027184
4	53	4.61	0.196349	0.118895	0.016799
11	45	4.82	0.291661	0.140054	0.031482
8	63	4.07	0.230502	0.116962	0.027904
6	59	4.70	0.194622	0.081616	0.024058
4	72	4.49	0.198502	0.090933	0.023960
6	71	4.21	0.311447	0.164807	0.034845
7	70	4.40	0.245016	0.115704	0.029416
7	71	4.44	0.234544	0.114722	0.026980
4	69	4.65	0.199155	0.087373	0.024047

13	62	4.65	0.170801	0.065692	0.022604
16	69	5.25	0.324762	0.151894	0.032951
13	71	4.72	0.331101	0.166638	0.034862
3	45	2.80	0.189227	0.104867	0.030095
19	35	3.13	0.163099	0.087800	0.024075
11	42	3.97	0.248218	0.126593	0.030636
17	35	3.55	0.182879	0.095426	0.024622
8	51	3.08	0.199204	0.113774	0.027734
6	51	3.62	0.228478	0.122009	0.029423
12	44	3.62	0.195920	0.102991	0.025697
6	57	3.63	0.125124	0.052529	0.019978
13	49	4.63	0.090629	0.024286	0.014344
6	56	4.19	0.104002	0.037846	0.015777
6	62	3.77	0.154326	0.067923	0.022892
8	71	3.58	0.265860	0.151224	0.032032
3	70	3.66	0.228449	0.117502	0.030337
13	68	3.78	0.213513	0.109761	0.027457
1	0	1.48	0.172142	0.125217	0.031686
1	1	1.98	0.220790	0.146880	0.037367
4	1	2.01	0.153505	0.105283	0.024025
1	12	2.15	0.192629	0.128389	0.029894
5	9	2.38	0.169621	0.105359	0.027026
2	14	3.05	0.194353	0.105893	0.029021
5	11	3.90	0.159495	0.074200	0.021883
17	13	2.47	0.184519	0.113853	0.028588
18	12	2.85	0.205905	0.121794	0.029478
12	22	2.65	0.162751	0.100529	0.023494
2	33	2.42	0.164294	0.100255	0.026482
8	25	2.79	0.095807	0.047466	0.017302
6	27	3.29	0.281776	0.166568	0.035014
13	20	2.77	0.088046	0.042046	0.016605
16	22	3.33	0.219694	0.123521	0.028849
18	20	4.17	0.126232	0.052015	0.017783
11	28	3.71	0.249479	0.134841	0.030862
15	21	2.96	0.158903	0.094225	0.021816
15	22	2.75	0.167000	0.099599	0.024539
16	21	2.84	0.175978	0.101871	0.026067
4	33	2.69	0.151511	0.097722	0.019994
8	35	2.67	0.146953	0.083846	0.023625
4	37	3.64	0.171116	0.089991	0.022287
8	33	3.73	0.204371	0.105565	0.026482
15	31	4.34	0.224701	0.100914	0.028503
12	28	2.63	0.162650	0.094814	0.025769
13	27	3.36	0.287051	0.168390	0.035276
6	34	2.38	0.119287	0.066987	0.021935
7	33	2.14	0.147288	0.092766	0.025445
13	33	2.72	0.187592	0.114344	0.026928
6	52	2.39	0.132145	0.076320	0.023328
5	53	2.67	0.125419	0.074754	0.018996
6	50	2.87	0.220075	0.131309	0.030890
13	50	3.23	0.232330	0.133311	0.030669
3	0	0.81	0.132204	0.109581	0.027805
2	1	0.95	0.160933	0.131910	0.030436
5	1	1.79	0.211055	0.153558	0.032083

7	0	2.11	0.159637	0.103396	0.026660
2	5	1.87	0.116284	0.079727	0.019598
1	13	1.49	0.165460	0.120695	0.030100
2	12	1.51	0.145618	0.106402	0.026027
1	14	2.37	0.198321	0.122563	0.031921
4	13	1.49	0.122269	0.091845	0.020376
5	16	1.45	0.060148	0.040372	0.013682
12	9	1.96	0.141950	0.098839	0.021992
3	22	2.05	0.161891	0.106817	0.026889
5	22	2.92	0.213994	0.125824	0.030206
16	11	3.65	0.161656	0.080680	0.022169
14	12	4.02	0.217274	0.107769	0.027250
14	11	1.82	0.110628	0.072492	0.020937
14	13	2.50	0.173500	0.107274	0.026452
5	20	1.44	0.091925	0.066676	0.017499
8	16	2.17	0.057184	0.029205	0.012866
2	22	3.09	0.198874	0.114765	0.027240
11	16	1.47	0.052287	0.033296	0.012950
6	20	2.11	0.084654	0.047499	0.017643
8	0	0.38	0.129158	0.118294	0.028661
4	4	0.69	0.108718	0.093788	0.021607
11	0	0.74	0.140137	0.118495	0.029212
12	0	0.60	0.115193	0.100830	0.023974
16	0	1.13	0.148689	0.115499	0.029499
15	1	1.25	0.151039	0.115627	0.028356
18	1	1.67	0.223475	0.165328	0.034847
18	0	0.41	0.140153	0.127885	0.030082
19	1	1.05	0.179250	0.144366	0.033143
5	15	1.46	0.116540	0.084042	0.022320
14	9	1.77	0.149185	0.103682	0.025781
2	21	1.84	0.141855	0.096550	0.024608
12	11	2.72	0.126722	0.077658	0.018066
14	15	0.73	0.098981	0.083775	0.020757
7	22	1.45	0.143305	0.106748	0.025270
3	25	1.23	0.095195	0.075507	0.016023
15	13	1.51	0.133730	0.098313	0.023455
8	20	2.12	0.087563	0.051858	0.016857
3	28	1.50	0.149061	0.105002	0.029411
11	20	1.43	0.080812	0.055734	0.017534
5	25	2.03	0.090648	0.056007	0.017033
6	24	1.21	0.056410	0.036660	0.016345
3	27	1.19	0.147282	0.113273	0.028614
19	20	1.09	0.077383	0.058253	0.017550
5	34	1.82	0.111517	0.072166	0.021578
6	33	2.30	0.175522	0.111576	0.027836
11	25	2.07	0.085989	0.050003	0.017405
14	22	2.90	0.205300	0.122565	0.028565
4	32	1.47	0.120769	0.085346	0.024059
17	20	1.36	0.094662	0.069089	0.018799
11	35	1.64	0.126380	0.086390	0.024399
3	44	2.56	0.185421	0.111762	0.028822
13	34	2.45	0.118573	0.065615	0.021599
18	27	1.10	0.207376	0.168869	0.035145
7	62	2.00	0.120875	0.077609	0.021604

11	58	2.64	0.183462	0.110633	0.027577
8	61	2.36	0.146354	0.088808	0.024417
5	59	2.65	0.143819	0.082061	0.023309
13	51	2.64	0.198657	0.121193	0.029345
13	52	2.84	0.138017	0.074154	0.022520
8	57	3.38	0.118083	0.054264	0.018884
8	58	3.45	0.203650	0.110430	0.027052
8	70	3.73	0.304058	0.175495	0.034448
11	34	0.97	0.087452	0.066578	0.021600
12	33	1.73	0.131734	0.091862	0.022988
7	44	2.16	0.169421	0.109018	0.027953
13	35	1.65	0.121871	0.081888	0.024281
8	46	1.97	0.071754	0.040043	0.016117
5	49	2.06	0.075597	0.044480	0.015080
15	37	1.99	0.152751	0.103892	0.024501
13	38	0.59	0.014833	0.009144	0.009648
18	33	1.48	0.152153	0.110847	0.027900
16	34	2.11	0.122747	0.074663	0.022762
5	45	0.90	0.166396	0.138349	0.031310
13	37	0.99	0.198566	0.164277	0.034611
17	33	1.13	0.137167	0.104600	0.028871
5	44	0.66	0.163145	0.141429	0.032680
14	35	0.93	0.110752	0.089429	0.022869
15	34	0.92	0.106711	0.088444	0.019848
11	46	1.43	0.066659	0.043095	0.016455
14	49	1.80	0.070046	0.045003	0.013929
8	55	2.52	0.151745	0.089317	0.024808
2	61	1.92	0.136008	0.089872	0.024058
5	56	2.64	0.092745	0.051846	0.015487
11	57	3.31	0.122572	0.057335	0.019723
11	52	1.32	0.106210	0.077244	0.021997
1	61	2.19	0.156081	0.095217	0.027729
8	54	1.36	0.193261	0.149631	0.032017
11	51	1.87	0.166642	0.114197	0.028062
5	57	1.25	0.089631	0.065639	0.019261
13	48	1.78	0.083640	0.050271	0.018769
16	45	2.09	0.189330	0.122412	0.032007
6	55	2.27	0.155015	0.095600	0.026120
16	44	1.27	0.173610	0.131960	0.032848
8	52	1.81	0.115639	0.075492	0.022156
13	55	1.53	0.135647	0.096545	0.025539
16	55	1.42	0.124145	0.086241	0.026637
12	59	1.88	0.117307	0.080774	0.019395
2	69	1.94	0.196110	0.137630	0.030221
11	54	1.69	0.204476	0.149411	0.032553
14	62	2.18	0.119004	0.072147	0.021506
6	73	1.92	0.229095	0.162828	0.034532
11	68	1.80	0.148825	0.100731	0.026706
14	59	2.74	0.140226	0.080779	0.021724
3	71	3.31	0.210163	0.116319	0.028337
11	63	2.99	0.200481	0.116820	0.027934
6	68	2.99	0.192543	0.108831	0.028034
5	69	3.34	0.275878	0.160973	0.034422
4	71	2.94	0.156975	0.089127	0.023059

15	69	2.63	0.174367	0.103671	0.026894
16	71	2.45	0.216837	0.138072	0.032112
11	70	2.71	0.265977	0.171765	0.034827
8	72	3.38	0.280553	0.165503	0.034017
12	71	4.13	0.213544	0.107956	0.025563
11	72	4.08	0.304040	0.163708	0.034403
14	69	3.80	0.279659	0.154931	0.032814
12	70	3.82	0.215950	0.110047	0.027689
11	71	2.81	0.243434	0.151381	0.032808
11	61	1.45	0.125116	0.089411	0.024606
1	3	-0.62	0.067395	0.082133	0.023713
5	0	0.15	0.123716	0.119190	0.029266
1	4	0.10	0.128413	0.125245	0.031821
3	9	0.23	0.114503	0.108642	0.025316
0	14	0.11	0.097646	0.094919	0.023950
8	6	-0.11	0.070726	0.073094	0.020888
14	1	0.56	0.164573	0.147310	0.030859
2	27	0.08	0.127529	0.125203	0.029533
7	28	0.94	0.124679	0.097621	0.028937
2	30	0.95	0.113053	0.089613	0.024611
7	25	1.89	0.098979	0.069240	0.015739
5	28	0.73	0.157971	0.135399	0.031131
18	18	0.21	0.061243	0.057242	0.018807
12	25	1.01	0.083453	0.068438	0.014871
13	24	1.80	0.061552	0.032693	0.016065
3	34	1.09	0.103835	0.080988	0.020960
15	27	1.40	0.137883	0.100572	0.026735
8	34	1.63	0.100112	0.065474	0.021257
15	28	0.86	0.115910	0.092278	0.027389
4	39	1.04	0.103765	0.081602	0.021234
6	37	1.42	0.214133	0.164616	0.034867
16	27	0.55	0.154197	0.136408	0.032182
18	22	0.64	0.164941	0.144844	0.031523
5	35	0.90	0.111844	0.090275	0.024004
16	25	1.33	0.088930	0.065108	0.017866
19	22	0.82	0.154953	0.131225	0.029110
6	35	1.24	0.116843	0.086013	0.024857
16	28	0.79	0.144405	0.119065	0.032130
17	28	-0.58	0.110424	0.129364	0.032556
8	37	0.09	0.154218	0.151427	0.032573
18	51	0.41	0.135978	0.123914	0.029584
8	62	0.94	0.086765	0.066140	0.021830
12	58	0.70	0.110529	0.093542	0.024295
14	56	0.49	0.059653	0.052542	0.014658
1	69	0.87	0.206338	0.174491	0.036767
18	57	1.40	0.085388	0.056560	0.020614
13	72	1.52	0.237906	0.183017	0.036086
15	72	1.41	0.143266	0.104706	0.027340
15	71	1.56	0.141808	0.101778	0.025681
16	70	2.03	0.216040	0.147793	0.033593
3	42	-0.61	0.094124	0.110763	0.027409
13	58	0.39	0.131205	0.120386	0.027842
19	52	1.17	0.102984	0.076594	0.022609
14	57	0.83	0.079973	0.064683	0.018322

6	58	0.73	0.139727	0.118733	0.028943
3	61	1.28	0.125649	0.094853	0.024038
8	56	2.03	0.072068	0.041163	0.015243
0	0	-1.65	0.071475	0.111321	0.024136
0	1	-1.42	0.065650	0.102880	0.026183
2	0	-1.03	0.081968	0.110188	0.027351
1	2	-0.94	0.076691	0.099557	0.024360
4	0	-1.39	0.075835	0.106773	0.022215
4	5	-0.96	0.084871	0.099495	0.015243
6	4	-0.69	0.115725	0.137267	0.031012
8	3	0.00	0.069773	0.069682	0.021532
7	4	-0.83	0.078436	0.100961	0.027027
2	9	-0.72	0.086926	0.104954	0.025178
4	8	-0.48	0.077164	0.088426	0.023283
13	6	-0.67	0.055607	0.069588	0.020724
17	1	-0.73	0.125062	0.149588	0.033760
8	10	-0.33	0.079749	0.087533	0.023642
16	4	0.00	0.116580	0.116556	0.030425
19	4	0.07	0.123771	0.121670	0.028491
13	10	0.30	0.101180	0.093846	0.024703
18	5	0.65	0.080837	0.066870	0.021496
18	4	0.53	0.153613	0.137606	0.030324
17	5	0.21	0.078470	0.074027	0.020918
16	9	1.18	0.140850	0.108560	0.027373
14	31	-0.71	0.111939	0.133095	0.029991
8	43	-0.61	0.103406	0.121280	0.029285
12	39	-0.41	0.083850	0.093576	0.023730
7	57	0.14	0.083046	0.080379	0.018800
3	62	0.49	0.095676	0.084750	0.022407
15	50	0.39	0.111218	0.100995	0.026097
19	46	0.65	0.056029	0.045297	0.016397
16	50	1.02	0.144431	0.113086	0.030684
7	59	1.41	0.111990	0.082316	0.021105
15	51	0.69	0.117660	0.100870	0.024195
5	62	1.44	0.105612	0.072704	0.022797
4	63	0.97	0.105956	0.085444	0.021065
6	61	1.03	0.119459	0.093278	0.025443
11	56	1.99	0.076527	0.045059	0.015830
5	71	0.84	0.173939	0.146378	0.032736
14	37	-0.87	0.113300	0.139802	0.030334
5	46	0.02	0.049901	0.049489	0.016679
4	44	0.12	0.087645	0.084901	0.022502
6	42	0.61	0.154265	0.134852	0.031667
5	50	0.86	0.147514	0.121604	0.030099
19	34	0.85	0.084613	0.066174	0.021619
5	43	-0.42	0.103840	0.116453	0.029790
13	42	0.54	0.152188	0.135399	0.031038
11	37	-0.57	0.132831	0.151479	0.032588
17	31	-0.78	0.109882	0.135575	0.033002
15	33	0.15	0.105458	0.101822	0.023471
16	39	-0.79	0.094139	0.116856	0.028718
1	51	0.12	0.128660	0.125074	0.029615
14	45	0.88	0.159352	0.132970	0.029893
8	50	0.55	0.137550	0.121561	0.029086

3	55	1.20	0.115348	0.084670	0.025662
11	41	-0.58	0.107487	0.123903	0.028451
18	34	-0.72	0.052874	0.069097	0.022436
11	48	-0.34	0.046681	0.053617	0.020381
15	44	-0.66	0.080820	0.097916	0.025864
13	46	0.12	0.035629	0.033661	0.016273
1	58	0.46	0.124284	0.109732	0.031438
7	50	0.10	0.100982	0.098134	0.027569
5	52	0.91	0.105304	0.085098	0.022207
13	43	0.09	0.138316	0.135491	0.030710
5	51	0.87	0.137039	0.112247	0.028561
5	58	-0.17	0.108432	0.113203	0.028311
12	51	0.44	0.105740	0.095710	0.022977
7	56	-0.08	0.070478	0.071648	0.014713
0	4	-1.99	0.045992	0.092596	0.023376
2	4	-1.11	0.078963	0.110071	0.028143
1	5	-1.13	0.057021	0.082418	0.022498
3	4	-0.91	0.081193	0.106544	0.027784
13	8	-1.04	0.117261	0.150228	0.031806
3	18	-0.53	0.082980	0.092372	0.017715
14	10	-0.23	0.078931	0.084237	0.022959
3	26	-1.00	0.077725	0.107190	0.029558
18	11	-0.74	0.056356	0.073475	0.023149
17	12	-0.71	0.097253	0.117486	0.028312
19	9	-0.65	0.085661	0.102637	0.026135
1	27	-0.81	0.128054	0.156422	0.034812
17	11	-0.50	0.067486	0.078938	0.023130
1	30	-0.80	0.048194	0.074762	0.033296
17	14	-0.27	0.110283	0.118783	0.030932
5	26	-1.03	0.111557	0.145339	0.032843
19	12	-0.41	0.101522	0.112745	0.027197
14	16	-0.01	0.041257	0.041337	0.012815
13	17	-0.15	0.010842	0.012248	0.009238
18	16	-0.49	0.023230	0.029958	0.013835
7	27	-0.32	0.103890	0.112751	0.027484
3	31	-0.34	0.101338	0.111718	0.030156
14	20	0.57	0.076743	0.067221	0.016643
1	33	-0.36	0.088249	0.099689	0.031906
0	35	0.25	0.132325	0.127187	0.020401
8	27	-0.26	0.143924	0.152328	0.032335
18	14	0.64	0.149849	0.129969	0.031100
16	16	0.30	0.060491	0.056482	0.013298
4	28	0.08	0.086896	0.084997	0.023272
17	15	-0.28	0.083211	0.089466	0.022490
13	19	0.14	0.007207	0.006174	0.007646
18	15	0.55	0.086743	0.074575	0.021984
2	31	0.96	0.145415	0.116249	0.030435
4	34	0.54	0.098862	0.089769	0.016744
14	25	0.97	0.071487	0.055529	0.016429
13	26	-1.00	0.141057	0.175433	0.034386
2	2	-2.29	0.048583	0.100469	0.022695
3	3	-1.47	0.052319	0.083210	0.021032
5	2	-1.64	0.050423	0.089755	0.023965
0	7	-1.73	0.044126	0.086109	0.024246

1	6	-1.10	0.052784	0.084800	0.029231
0	8	-1.35	0.054392	0.086657	0.023951
5	3	-2.10	0.028166	0.074819	0.022207
6	2	-1.25	0.049105	0.077549	0.022714
1	7	-2.23	0.055462	0.130584	0.033746
2	6	-2.24	0.046563	0.095384	0.021830
9	0	-2.21	0.047669	0.106591	0.026689
1	8	-2.19	0.059718	0.135732	0.034715
6	3	-1.59	0.036567	0.071806	0.022142
5	4	-1.12	0.093014	0.126057	0.029595
7	3	-1.80	0.039570	0.076398	0.020452
8	2	-1.42	0.046723	0.078535	0.022417
1	9	-1.44	0.075872	0.117651	0.029053
4	7	-1.65	0.050365	0.087749	0.022644
8	4	-1.29	0.089399	0.127245	0.029387
1	11	-0.81	0.068147	0.087186	0.023565
6	6	-0.86	0.052622	0.071765	0.022213
14	0	-1.32	0.079359	0.115804	0.027707
11	3	-0.77	0.054344	0.071206	0.021915
13	2	-0.84	0.055935	0.074631	0.022183
4	11	-0.63	0.079850	0.089977	0.016049
5	10	-0.97	0.061470	0.085218	0.024493
17	0	-0.82	0.096262	0.120342	0.029475
13	4	-0.49	0.125368	0.140145	0.030073
2	15	-0.37	0.093516	0.101448	0.021427
8	8	-0.30	0.127679	0.136930	0.030612
7	9	0.15	0.105526	0.101872	0.024339
6	10	-0.11	0.091243	0.093878	0.024709
15	4	0.11	0.103573	0.100807	0.025799
9	10	-2.05	0.031744	0.077015	0.022036
10	8	-2.29	0.052400	0.119681	0.029413
9	13	-1.50	0.062556	0.099756	0.024806
19	6	-1.11	0.044192	0.071488	0.024520
15	9	-0.37	0.105336	0.114096	0.023769
18	6	-0.81	0.054314	0.073322	0.023378
19	5	-1.17	0.043369	0.066764	0.020015
18	9	-0.22	0.103288	0.109471	0.028360
1	28	-2.06	0.051072	0.123039	0.034936
6	23	-1.73	0.053964	0.098006	0.025407
5	24	-1.30	0.027145	0.048916	0.016804
19	10	-2.14	0.038709	0.087862	0.022975
0	29	-2.21	0.033265	0.080942	0.021583
12	16	-1.62	0.049655	0.067687	0.011155
0	28	-1.55	0.038364	0.078985	0.026224
9	22	-2.20	0.052801	0.110150	0.026033
8	23	-2.20	0.037310	0.091227	0.024532
2	29	-1.36	0.090904	0.128112	0.027322
2	28	-0.41	0.098681	0.110966	0.029861
2	32	-1.44	0.084307	0.128768	0.030781
5	30	-0.52	0.085998	0.097916	0.022840
19	16	-0.84	0.027597	0.038232	0.012606
17	18	-0.96	0.052491	0.070528	0.018869
3	29	-0.81	0.091633	0.112587	0.025730
19	14	-0.21	0.110100	0.116038	0.028525

7	26	0.00	0.109348	0.109296	0.027668
8	30	0.11	0.089344	0.086645	0.023628
3	32	-1.95	0.058357	0.116911	0.030099
1	34	-0.96	0.052356	0.076173	0.024908
6	29	-0.92	0.134942	0.166138	0.034054
6	26	-1.01	0.138025	0.172585	0.034281
8	24	-0.55	0.030495	0.039182	0.015864
5	27	-0.03	0.143413	0.144477	0.032530
3	30	-1.22	0.070798	0.100125	0.023972
17	16	-0.64	0.038561	0.047225	0.013479
4	29	-0.91	0.061958	0.081259	0.021317
10	28	-1.14	0.076877	0.112319	0.030960
12	26	-1.38	0.068389	0.103562	0.025424
17	22	-0.41	0.120638	0.132777	0.029955
1	37	-1.56	0.108978	0.162729	0.034539
2	37	-1.47	0.088665	0.130118	0.028265
12	27	-0.65	0.090130	0.106714	0.025689
8	31	0.01	0.141353	0.140885	0.031421
18	21	-1.01	0.078758	0.107501	0.028538
6	30	-0.51	0.077646	0.090296	0.024630
5	31	-0.68	0.116658	0.138180	0.031516
16	20	-0.02	0.079558	0.079967	0.018946
2	34	-0.93	0.055228	0.074747	0.021017
6	31	-0.54	0.136285	0.154204	0.033237
14	28	-0.58	0.113226	0.130274	0.029507
7	35	0.11	0.098389	0.095859	0.022189
11	31	-0.54	0.122723	0.139756	0.031584
14	26	-0.90	0.113620	0.141478	0.031098
3	37	-0.34	0.109027	0.117956	0.026403
19	21	-0.87	0.074875	0.097800	0.026269
11	30	-0.23	0.080120	0.085867	0.025347
14	27	0.22	0.147233	0.140222	0.031313
2	39	-0.85	0.085767	0.108684	0.026908
2	44	-0.51	0.105894	0.121284	0.029938
4	42	-0.54	0.086650	0.098464	0.021880
17	30	0.13	0.089520	0.086005	0.027317
12	34	-0.91	0.056030	0.072820	0.018400
16	23	-2.01	0.034152	0.085259	0.025380
7	32	-1.21	0.078128	0.113636	0.029257
13	23	-1.16	0.069953	0.099296	0.025375
19	18	-1.62	0.033735	0.062228	0.017607
8	29	-1.47	0.105685	0.152348	0.031697
17	25	-0.89	0.043171	0.059566	0.018501
13	29	-1.35	0.118970	0.166174	0.034921
13	30	-0.47	0.081583	0.092327	0.022643
18	25	-0.19	0.045886	0.049475	0.018747
8	39	-1.04	0.096628	0.126625	0.028798
5	42	-0.56	0.108701	0.125383	0.029857
12	35	-0.35	0.088339	0.095369	0.020170
16	31	-0.51	0.109677	0.126055	0.032113
15	32	0.45	0.110425	0.097831	0.027797
8	36	-1.91	0.013276	0.037353	0.012631
17	27	-1.20	0.110883	0.150112	0.032791
6	38	-0.69	0.010546	0.017212	0.009633

13	31	-0.40	0.144223	0.157425	0.032689
4	47	-0.91	0.062205	0.082876	0.022722
17	34	-0.91	0.051280	0.072274	0.023084
7	43	-0.56	0.073660	0.088450	0.026457
11	39	-0.61	0.107897	0.125677	0.029297
18	37	-0.91	0.134419	0.166429	0.035181
8	47	-0.03	0.141484	0.142498	0.031414
3	52	-0.15	0.096184	0.099377	0.021687
2	53	0.74	0.102393	0.087748	0.019763
4	51	0.05	0.096617	0.095591	0.020847
6	48	0.99	0.071500	0.051587	0.020156
11	43	-0.12	0.116430	0.120045	0.029626
12	42	-0.40	0.091722	0.101383	0.024360
3	51	-0.12	0.099159	0.102272	0.025954
13	41	-0.09	0.127939	0.130784	0.030839
5	48	-0.03	0.062631	0.063265	0.019510
3	50	-0.08	0.102683	0.104981	0.028492
6	46	0.47	0.045823	0.037887	0.016715
7	63	-1.21	0.066761	0.095990	0.024131
5	65	-1.25	0.038122	0.064682	0.021307
15	55	-1.01	0.062664	0.087658	0.024666
18	52	-0.71	0.061652	0.078347	0.023460
12	53	-0.59	0.078754	0.089228	0.017882
14	51	0.04	0.111647	0.110608	0.027401
5	61	0.19	0.098906	0.094220	0.024661
3	73	0.37	0.130178	0.120103	0.027240
6	60	-0.81	0.114711	0.140403	0.031852
14	53	0.04	0.074313	0.073589	0.018548
11	65	-0.10	0.058020	0.060075	0.020313
8	68	0.79	0.122277	0.101211	0.026654
19	57	1.45	0.088448	0.059215	0.020217
16	60	0.34	0.127280	0.117130	0.029423
4	73	1.04	0.115122	0.091660	0.022532
5	72	0.38	0.169307	0.156600	0.033460
16	61	0.75	0.112149	0.092536	0.026314
6	72	1.64	0.239851	0.180630	0.036185
16	62	1.01	0.102012	0.078242	0.023492
7	72	-0.34	0.109768	0.119543	0.028558
18	61	-0.21	0.089419	0.094787	0.026068
12	61	0.42	0.094113	0.085224	0.021131
16	57	1.12	0.099160	0.076004	0.020592
13	61	1.37	0.126801	0.092999	0.024609
18	56	1.00	0.058581	0.042595	0.015913
14	71	-0.01	0.139889	0.140323	0.031396
11	73	-0.03	0.149154	0.149997	0.032321
14	70	0.86	0.186974	0.158708	0.032870
13	66	-1.06	0.054289	0.078143	0.022544
14	58	-0.52	0.095736	0.109605	0.026642
2	71	0.46	0.140427	0.126973	0.029354
17	55	-0.60	0.073560	0.089966	0.027327
19	53	0.38	0.070825	0.062496	0.022206
16	56	0.34	0.069273	0.063731	0.016249
18	55	-0.78	0.076165	0.096410	0.025873
5	68	-0.12	0.097943	0.101092	0.027292

2	72	-0.50	0.119575	0.134629	0.029967
4	70	-0.61	0.065976	0.081135	0.025054
14	60	-0.39	0.107713	0.119320	0.029589
3	72	0.38	0.132620	0.121371	0.029429
5	70	0.91	0.195940	0.164719	0.034166
8	67	-0.12	0.136465	0.140194	0.031094
19	56	0.84	0.060337	0.047596	0.015086
12	72	-0.51	0.099524	0.112959	0.026356
16	68	-0.31	0.086006	0.094418	0.027500
18	69	0.47	0.205799	0.187889	0.037878
13	73	0.82	0.190999	0.162863	0.034110
8	73	0.72	0.173277	0.150175	0.032173
13	67	0.02	0.152523	0.151747	0.033107
9	35	-2.25	0.044606	0.093650	0.021804
12	32	-1.61	0.064528	0.107766	0.026876
3	41	-1.20	0.075157	0.103061	0.023292
15	29	-1.07	0.071729	0.096827	0.023554
17	42	-1.30	0.086561	0.127531	0.031440
12	47	-1.26	0.069148	0.100492	0.024929
4	55	-1.31	0.061229	0.086909	0.019647
7	52	-1.12	0.066096	0.089546	0.020942
5	54	-0.74	0.119962	0.143568	0.031716
14	44	-0.81	0.110980	0.136359	0.031369
16	42	-0.14	0.116528	0.120873	0.030182
7	51	0.58	0.113276	0.098556	0.025240
11	47	-0.66	0.121881	0.142307	0.031168
4	54	-0.72	0.069461	0.085494	0.022175
1	57	-0.90	0.056456	0.077414	0.023182
14	43	-1.10	0.081863	0.112998	0.028238
8	48	-0.18	0.048698	0.052048	0.019064
19	37	-0.35	0.138909	0.150153	0.032200
7	49	-0.45	0.058546	0.065290	0.014901
19	44	-0.44	0.128007	0.141798	0.031675
3	60	-0.65	0.081151	0.099148	0.027844
12	50	-0.37	0.086665	0.095796	0.024551
7	55	-0.61	0.062290	0.077286	0.024390
7	54	-0.31	0.102155	0.110635	0.027732
10	51	-0.70	0.088238	0.107101	0.027003
19	49	0.23	0.043023	0.039437	0.015322
7	61	0.58	0.099694	0.086349	0.023128
16	52	1.08	0.114566	0.089538	0.023105
15	53	1.27	0.140175	0.115395	0.019554
10	34	-2.44	0.023775	0.077007	0.021795
5	40	-2.26	0.069494	0.145791	0.033718
1	44	-2.01	0.078069	0.148709	0.035144
0	45	-2.02	0.026363	0.079602	0.026340
2	43	-1.77	0.048960	0.098638	0.028068
6	39	-1.23	0.098682	0.136439	0.030686
13	32	-2.18	0.107658	0.185423	0.035696
19	32	-1.93	0.092367	0.156341	0.033200
1	49	-1.30	0.034962	0.057868	0.017572
19	31	-1.24	0.094378	0.133317	0.031451
19	30	-0.98	0.047906	0.074966	0.027578
6	43	-0.08	0.131110	0.133544	0.031132

1	48	-1.38	0.025413	0.061340	0.025956
9	39	-1.91	0.054851	0.104155	0.025770
2	46	-1.51	0.038767	0.062736	0.015853
1	47	-1.80	0.088050	0.147927	0.033310
16	32	-1.31	0.097341	0.141231	0.033473
7	41	-0.88	0.084244	0.104423	0.022866
14	34	-1.00	0.050624	0.070745	0.020145
7	48	-0.44	0.058923	0.068341	0.021175
9	46	-1.64	0.034580	0.059965	0.015488
19	36	-1.90	0.013870	0.044496	0.016084
1	54	-1.13	0.111564	0.149898	0.033940
4	50	-0.70	0.081324	0.096809	0.021970
15	39	-0.96	0.069225	0.091902	0.023736
7	47	-1.36	0.070031	0.105805	0.026347
18	36	-1.67	0.009957	0.034772	0.014867
1	53	-1.61	0.038571	0.081564	0.026769
0	54	-1.73	0.041780	0.082366	0.023470
14	39	-1.33	0.083199	0.119514	0.027407
2	50	-0.75	0.085961	0.107344	0.028620
6	47	-1.76	0.096871	0.156717	0.033970
1	52	-1.34	0.056149	0.091061	0.025961
2	51	-0.93	0.080882	0.105468	0.026382
12	41	-0.78	0.080392	0.097360	0.021788
3	49	-1.01	0.057773	0.073443	0.015484
0	3	-3.52	0.036573	0.100241	0.018105
2	3	-2.74	0.020864	0.078019	0.020890
3	2	-2.94	0.042608	0.112585	0.023840
7	2	-3.08	0.030890	0.101207	0.022846
2	7	-2.14	0.051406	0.111311	0.028030
3	6	-2.20	0.062979	0.109795	0.021270
10	0	-2.25	0.047521	0.112657	0.029012
2	8	-2.12	0.056007	0.117373	0.028891
9	1	-2.47	0.054238	0.127385	0.029591
3	7	-2.19	0.046084	0.105915	0.027286
0	10	-2.88	0.020010	0.075131	0.019143
3	8	-2.20	0.051665	0.112214	0.027484
5	8	-2.86	0.047679	0.134937	0.030553
10	3	-2.17	0.032756	0.080143	0.021839
9	4	-2.02	0.052409	0.106520	0.026834
11	2	-1.03	0.057862	0.081508	0.022920
0	13	-1.90	0.053986	0.094760	0.021406
2	11	-1.16	0.055935	0.079980	0.020729
6	7	-2.24	0.069939	0.141029	0.031738
3	10	-1.43	0.042795	0.076144	0.023381
7	6	-2.74	0.031538	0.094163	0.022866
6	8	-1.81	0.089685	0.148006	0.032300
10	4	-2.46	0.041119	0.112196	0.028914
7	7	-2.32	0.038432	0.100794	0.026894
4	10	-2.33	0.029447	0.073578	0.018934
9	5	-1.86	0.041265	0.076060	0.018719
15	0	-1.83	0.062037	0.111022	0.026730
7	8	-1.50	0.063599	0.105440	0.027859
11	4	-0.81	0.103142	0.126848	0.029389
14	3	-2.20	0.028091	0.074293	0.020964

1	16	-2.07	0.031599	0.061440	0.014382
10	7	-2.27	0.047756	0.113580	0.029054
7	10	-1.42	0.042295	0.073524	0.021963
11	6	-1.10	0.050649	0.075421	0.022473
13	3	-1.75	0.031664	0.070073	0.021912
12	4	-1.12	0.070150	0.097613	0.024481
1	15	-1.76	0.062552	0.103810	0.023388
19	0	-0.84	0.092560	0.116243	0.028353
11	8	-0.59	0.117469	0.135969	0.031166
14	2	-2.11	0.043871	0.089555	0.021680
15	3	-2.72	0.037380	0.091419	0.019849
18	3	-1.73	0.035603	0.074884	0.022668
17	4	-0.78	0.100513	0.124653	0.030801
19	2	-1.31	0.054852	0.083024	0.021586
9	12	-1.53	0.063557	0.101438	0.024711
11	10	-0.58	0.073230	0.087446	0.024398
2	16	-2.83	0.026840	0.061510	0.012271
14	4	-1.89	0.068579	0.121491	0.027969
16	2	-2.31	0.039035	0.096621	0.024924
9	9	-1.87	0.055819	0.101657	0.024497
11	7	-2.11	0.064610	0.129835	0.030857
17	3	-2.11	0.028272	0.076683	0.022989
18	2	-1.71	0.043692	0.082911	0.022992
1	22	-1.49	0.096045	0.142919	0.031485
3	20	-0.91	0.084861	0.100992	0.017749
15	7	-2.17	0.039369	0.095905	0.026009
1	21	-1.52	0.063495	0.108379	0.029446
12	10	-0.80	0.056015	0.072148	0.020282
16	6	-1.68	0.052743	0.092794	0.023850
6	19	-0.92	0.006478	0.014287	0.008504
2	23	-0.93	0.059602	0.081710	0.023881
1	24	-1.59	0.032340	0.062166	0.018705
10	14	-1.52	0.061697	0.106965	0.029831
0	9	-3.99	0.033682	0.118258	0.021183
4	6	-3.70	0.056554	0.126748	0.018978
10	1	-3.14	0.036236	0.135108	0.031502
9	2	-3.16	0.027948	0.097457	0.022019
1	10	-2.81	0.025634	0.093791	0.024298
5	6	-2.68	0.032586	0.087333	0.020422
9	3	-2.59	0.023076	0.075670	0.020271
5	7	-2.13	0.063107	0.127833	0.030324
0	12	-1.97	0.060789	0.101425	0.020680
10	2	-3.16	0.027058	0.102041	0.023720
2	10	-2.80	0.016035	0.078879	0.022447
12	2	-3.36	0.035387	0.103730	0.020351
12	3	-2.76	0.025812	0.076598	0.018368
8	7	-2.59	0.050486	0.130064	0.030730
9	6	-3.15	0.025483	0.091074	0.020826
10	5	-3.29	0.017127	0.082083	0.019758
9	8	-2.49	0.043987	0.112502	0.027549
9	7	-2.69	0.033034	0.107716	0.027764
15	2	-3.71	0.049407	0.132662	0.022452
10	6	-3.09	0.029500	0.099153	0.022527
16	3	-2.43	0.023408	0.078731	0.022770

3	16	-1.75	0.049950	0.073181	0.013297
17	2	-1.95	0.043892	0.091393	0.024323
10	9	-2.41	0.044070	0.107651	0.026392
12	7	-2.18	0.041865	0.096095	0.024893
1	18	-1.47	0.052718	0.082310	0.020064
12	6	-3.60	0.022375	0.095758	0.020406
14	7	-2.61	0.047363	0.122775	0.028868
10	11	-2.05	0.038325	0.082365	0.021500
0	21	-3.37	0.021492	0.090721	0.020563
1	20	-2.75	0.023149	0.079719	0.020558
12	8	-1.78	0.055205	0.100586	0.025548
13	7	-2.71	0.058482	0.142772	0.031050
2	18	-2.47	0.037986	0.081775	0.017755
9	11	-2.10	0.034579	0.075965	0.019718
10	10	-2.39	0.023967	0.079773	0.023359
14	6	-2.35	0.038481	0.085249	0.019881
15	5	-1.46	0.072862	0.099097	0.017987
15	8	-1.32	0.064960	0.099338	0.026136
9	14	-2.67	0.028017	0.101918	0.027638
7	16	-2.11	0.041806	0.067148	0.012025
0	23	-2.62	0.027044	0.081263	0.020664
10	13	-1.86	0.057330	0.105868	0.026153
16	7	-1.82	0.065040	0.120320	0.030398
6	17	-0.91	0.012277	0.020956	0.009519
5	18	-2.46	0.021009	0.065772	0.018212
17	6	-2.01	0.034247	0.083379	0.024424
19	3	-1.56	0.038445	0.071414	0.021119
14	8	-2.72	0.050241	0.129396	0.029127
0	22	-2.09	0.044713	0.090860	0.022038
5	17	-3.05	0.018005	0.048431	0.009984
10	12	-2.79	0.034628	0.109439	0.026770
17	8	-2.63	0.050158	0.135647	0.032518
19	8	-2.22	0.060136	0.130340	0.031635
2	25	-1.80	0.037343	0.067482	0.016776
8	19	-2.00	0.007497	0.024404	0.008443
7	20	-1.85	0.057569	0.089935	0.017476
1	25	-1.00	0.047392	0.067837	0.020503
15	6	-4.07	0.030267	0.121667	0.022475
9	16	-3.31	0.018945	0.058460	0.011943
10	15	-2.72	0.044494	0.104986	0.022251
18	7	-2.64	0.054943	0.142154	0.033060
8	17	-1.99	0.012109	0.030404	0.009192
15	10	-2.36	0.023339	0.075847	0.022244
16	8	-1.44	0.081696	0.126317	0.031047
1	23	-2.42	0.027020	0.091325	0.026598
17	7	-1.97	0.066642	0.128838	0.031544
9	15	-3.63	0.025094	0.096952	0.019787
1	26	-2.87	0.063064	0.157119	0.032800
4	23	-2.88	0.025556	0.080631	0.019146
3	24	-1.94	0.040828	0.073438	0.016794
0	27	-1.23	0.056599	0.085707	0.023711
9	18	-2.30	0.038739	0.077945	0.017028
17	10	-2.11	0.037099	0.088272	0.024211
18	8	-1.77	0.088762	0.147970	0.033471

17	9	-1.34	0.072058	0.109538	0.028069
3	23	-0.87	0.060167	0.081414	0.024431
0	26	-2.39	0.020705	0.076305	0.023289
2	24	-1.83	0.033637	0.063024	0.016090
19	7	-2.30	0.054565	0.125320	0.030813
15	11	-1.62	0.062192	0.092087	0.018505
16	10	-1.25	0.053209	0.083103	0.023960
5	19	-4.02	0.011165	0.043692	0.008094
10	16	-3.94	0.013443	0.063866	0.012793
9	20	-3.29	0.027368	0.082219	0.016652
2	26	-2.32	0.053986	0.122054	0.029300
5	23	-2.40	0.029409	0.090788	0.025526
10	18	-2.37	0.040434	0.084566	0.018628
11	17	-1.93	0.018983	0.037029	0.009336
18	10	-1.62	0.055453	0.096150	0.025144
7	24	-2.50	0.024752	0.065833	0.016421
0	31	-2.33	0.029696	0.090582	0.026097
10	21	-1.39	0.065010	0.100301	0.025404
7	23	-1.80	0.032305	0.075085	0.023713
9	21	-1.37	0.059946	0.092134	0.023534
19	11	-2.39	0.021079	0.071956	0.021286
1	29	-2.36	0.085482	0.165132	0.033703
19	15	-1.57	0.046610	0.078957	0.020581
9	25	-2.27	0.027675	0.063568	0.015778
8	26	-1.68	0.102545	0.156867	0.032415
16	18	-0.95	0.059660	0.077828	0.019106
11	23	-2.13	0.037299	0.090685	0.025062
10	24	-2.12	0.029244	0.065064	0.016914
10	29	-2.66	0.056178	0.129187	0.027426
10	26	-2.18	0.056996	0.122741	0.030107
12	24	-2.63	0.028519	0.067469	0.014834
9	27	-2.18	0.058357	0.120523	0.028522
7	29	-1.74	0.072888	0.115642	0.024536
7	30	-1.93	0.031079	0.081926	0.026409
9	28	-2.48	0.037469	0.107721	0.028345
18	19	-1.69	0.006168	0.022095	0.009452
11	26	-2.06	0.088914	0.155667	0.032435
0	37	-2.35	0.036679	0.090152	0.022752
14	23	-2.04	0.041181	0.089057	0.023511
14	17	-3.74	0.019339	0.051428	0.008577
0	30	-3.16	0.026804	0.105632	0.024938
15	15	-3.10	0.075960	0.139168	0.020370
11	19	-2.48	0.008248	0.030521	0.008969
4	26	-1.84	0.039190	0.081144	0.022861
10	20	-3.66	0.021740	0.089008	0.018373
5	29	-3.01	0.051388	0.143346	0.030522
10	25	-2.49	0.027577	0.069620	0.016876
9	26	-1.76	0.068728	0.118747	0.028385
12	23	-1.83	0.036097	0.075996	0.021862
11	24	-1.23	0.022279	0.042406	0.016419
18	17	-2.44	0.007494	0.029539	0.009053
10	22	-2.18	0.055533	0.116118	0.027733
9	23	-2.31	0.026057	0.079764	0.023220
19	13	-1.34	0.073087	0.110022	0.027482

14	18	-1.12	0.046441	0.066037	0.017527
1	31	-2.02	0.064400	0.133780	0.034425
0	32	-2.16	0.023675	0.078041	0.025178
12	20	-2.76	0.049318	0.092516	0.015668
10	23	-2.16	0.028117	0.083016	0.025367
9	24	-2.34	0.023669	0.060497	0.015706
1	32	-2.46	0.066066	0.156181	0.036629
0	33	-2.82	0.032264	0.098255	0.023361
19	19	-2.79	0.008542	0.036524	0.010034
15	23	-2.49	0.022930	0.081534	0.023512
14	24	-1.52	0.025967	0.049678	0.015576
11	27	-1.04	0.117326	0.151091	0.032514
7	31	-2.00	0.046349	0.104711	0.029154
17	21	-1.12	0.074009	0.104097	0.026897
5	33	-0.84	0.089945	0.112017	0.026390
6	32	-2.04	0.109162	0.181685	0.035589
9	29	-1.91	0.070759	0.122278	0.027010
9	30	-1.43	0.051735	0.086844	0.024579
0	39	-2.44	0.025796	0.079194	0.021886
1	36	-2.82	0.013552	0.068626	0.019533
10	27	-2.64	0.047631	0.126861	0.030005
5	32	-2.18	0.083936	0.156653	0.033362
12	30	-2.23	0.031161	0.083960	0.023655
2	40	-2.11	0.055206	0.120762	0.031053
3	39	-1.71	0.060042	0.103266	0.025306
16	26	-1.93	0.073026	0.134016	0.031665
5	37	-1.35	0.102119	0.145078	0.031781
19	23	-1.65	0.048626	0.088642	0.024270
18	24	-0.94	0.025211	0.041137	0.017013
6	36	-1.14	0.014212	0.029387	0.013285
0	42	-2.65	0.038290	0.100900	0.023632
10	32	-2.88	0.039522	0.130501	0.031583
1	41	-2.59	0.061361	0.139285	0.030136
9	33	-1.76	0.052552	0.097076	0.025265
3	40	-1.96	0.049586	0.108039	0.029780
0	43	-1.27	0.046142	0.074986	0.022798
17	26	-2.37	0.073090	0.150837	0.032788
19	24	-1.88	0.014163	0.045006	0.016437
1	42	-1.22	0.092511	0.132976	0.033071
5	38	-1.79	0.023375	0.041807	0.010298
12	31	-1.42	0.064154	0.101806	0.026547
11	32	-0.83	0.134479	0.162544	0.033849
2	41	-1.60	0.072377	0.111913	0.024783
9	34	-2.15	0.028341	0.071602	0.020098
7	34	-1.20	0.047548	0.072760	0.020924
16	30	-0.50	0.076956	0.090395	0.026695
10	33	-2.24	0.041665	0.103673	0.027652
17	23	-1.96	0.039793	0.090454	0.025858
16	24	-1.92	0.026346	0.059788	0.017411
14	29	-2.96	0.049804	0.138784	0.030057
10	30	-2.13	0.039731	0.093462	0.025280
0	40	-1.89	0.031499	0.076103	0.023562
1	39	-2.48	0.046496	0.125785	0.031992
9	31	-2.29	0.046425	0.112814	0.029024

8	32	-1.31	0.121134	0.164800	0.033285
11	29	-2.18	0.083138	0.152665	0.031956
1	40	-2.91	0.054827	0.152229	0.033519
15	26	-2.00	0.039764	0.093467	0.026816
17	24	-1.13	0.033055	0.052676	0.017414
18	23	-2.17	0.042684	0.099280	0.026043
1	45	-2.06	0.058142	0.129118	0.034512
3	43	-1.23	0.057420	0.092395	0.028512
7	39	-0.26	0.091551	0.098208	0.025351
9	37	-1.85	0.072425	0.124066	0.027853
19	27	-1.20	0.110994	0.149013	0.031719
8	38	-2.05	0.007431	0.025650	0.008873
6	41	-1.19	0.096043	0.132435	0.030660
15	24	-3.96	0.023372	0.091206	0.017121
17	19	-3.68	0.012372	0.049927	0.010218
5	36	-3.36	0.016380	0.055696	0.011710
10	31	-2.84	0.030518	0.118308	0.030897
9	32	-1.97	0.064876	0.124684	0.030408
12	29	-1.87	0.062618	0.107344	0.023936
0	41	-2.98	0.027325	0.085563	0.019511
6	40	-2.24	0.092677	0.170774	0.034892
18	28	-1.59	0.096743	0.148635	0.032538
14	32	-2.98	0.055664	0.150764	0.031872
5	41	-2.64	0.045363	0.118861	0.027875
17	29	-2.56	0.070382	0.152852	0.032161
7	40	-2.24	0.043055	0.107206	0.028630
19	28	-1.88	0.069644	0.127542	0.030825
2	45	-1.83	0.056083	0.111631	0.030324
4	43	-2.59	0.023885	0.078215	0.020995
10	37	-2.20	0.069459	0.132147	0.028485
1	46	-1.90	0.026665	0.061841	0.018506
0	47	-2.07	0.033331	0.081701	0.023358
11	36	-2.15	0.012494	0.041533	0.013496
14	33	-1.32	0.073111	0.106894	0.025583
18	29	-1.54	0.114804	0.169383	0.035367
14	30	-1.09	0.069393	0.094135	0.022748
1	43	-0.65	0.099110	0.118916	0.030473
19	25	-1.04	0.032974	0.051420	0.017721
4	40	-2.11	0.033003	0.081890	0.023207
0	44	-1.65	0.042810	0.082771	0.024173
5	39	-1.20	0.089851	0.124168	0.028600
7	37	-0.27	0.111668	0.118563	0.025562
2	42	-1.14	0.082032	0.113471	0.027608
18	26	-3.39	0.057305	0.174740	0.034638
19	26	-3.21	0.053898	0.154090	0.031212
10	35	-2.49	0.042157	0.099797	0.023152
4	41	-2.39	0.036229	0.082876	0.019528
16	29	-1.95	0.081651	0.139678	0.029717
3	48	-1.79	0.044634	0.080601	0.020129
1	50	-1.92	0.058766	0.121234	0.032492
2	49	-1.98	0.032288	0.061421	0.014703
10	41	-2.41	0.052146	0.113927	0.025605
2	48	-2.00	0.028551	0.069252	0.020352
10	40	-2.33	0.048577	0.121294	0.031271

4	45	-1.57	0.047077	0.085377	0.024337
12	37	-1.35	0.077245	0.110508	0.024582
7	42	-0.99	0.079383	0.105550	0.026380
2	47	-1.01	0.089058	0.118126	0.028900
13	36	-0.59	0.015936	0.022758	0.011622
11	38	-0.54	0.026418	0.031398	0.009182
18	31	-0.75	0.128703	0.153836	0.033418
8	41	-0.67	0.103470	0.122362	0.028364
18	30	-0.99	0.060185	0.086696	0.026676
2	67	-2.38	0.054442	0.120511	0.027733
19	50	-1.65	0.068869	0.117044	0.029127
6	63	-2.23	0.061000	0.125810	0.029059
14	55	-1.47	0.053888	0.089625	0.024239
16	53	-1.58	0.050167	0.084557	0.021791
15	54	-1.54	0.060019	0.098813	0.025235
0	69	-1.10	0.056906	0.084116	0.024669
12	57	-0.24	0.076719	0.080726	0.016982
5	66	-1.50	0.045235	0.080421	0.023496
18	53	-0.67	0.046781	0.061494	0.021834
11	60	-0.84	0.102382	0.128510	0.030951
16	48	-1.25	0.038922	0.067156	0.022637
14	50	-0.47	0.104265	0.117588	0.028179
11	40	-3.42	0.041058	0.153044	0.032727
9	42	-2.77	0.034384	0.109662	0.027211
0	51	-3.07	0.032633	0.098261	0.021393
0	50	-3.03	0.024270	0.096664	0.023897
4	46	-2.45	0.059255	0.094110	0.014252
3	47	-1.75	0.056938	0.105823	0.027945
18	32	-2.11	0.103790	0.180639	0.036353
9	40	-2.64	0.038295	0.117042	0.029873
10	39	-2.58	0.039637	0.110812	0.027638
3	46	-1.70	0.044482	0.072906	0.016702
17	32	-2.58	0.067065	0.156764	0.034782
8	40	-3.25	0.046500	0.153962	0.033080
19	29	-2.39	0.077424	0.152762	0.031561
15	40	-2.13	0.037129	0.094818	0.027056
10	45	-1.77	0.058640	0.113193	0.030872
12	43	-0.80	0.066749	0.085966	0.024051
0	55	-2.68	0.029240	0.089302	0.022397
14	41	-1.99	0.061247	0.115328	0.027129
14	40	-1.79	0.083829	0.140650	0.031720
10	44	-2.82	0.037001	0.123158	0.030530
9	45	-2.02	0.051050	0.109074	0.028663
17	37	-1.56	0.101402	0.152383	0.032623
2	52	-2.80	0.030021	0.090188	0.021456
13	40	-2.77	0.078473	0.173702	0.034440
9	44	-1.79	0.064887	0.116414	0.028851
10	43	-0.95	0.071625	0.099366	0.029162
16	37	-1.87	0.083930	0.140920	0.030489
7	46	-1.60	0.039429	0.065534	0.016327
17	36	-2.49	0.019537	0.058368	0.015578
9	43	-2.23	0.036010	0.096333	0.027001
13	39	-1.70	0.086450	0.138407	0.030639
10	42	-1.79	0.064547	0.115887	0.028731

5	47	-1.26	0.096284	0.135986	0.031468
14	38	-2.56	0.020409	0.044354	0.009349
19	33	-1.60	0.054505	0.097760	0.027044
19	40	-2.49	0.072360	0.150629	0.031496
16	43	-1.73	0.055040	0.106195	0.029625
9	50	-1.41	0.065539	0.103971	0.027272
16	47	-1.72	0.075389	0.128648	0.030930
4	59	-1.58	0.062200	0.090964	0.018213
3	59	-0.83	0.068863	0.087267	0.022187
17	44	-0.28	0.134764	0.144184	0.033722
11	50	0.41	0.133044	0.120884	0.029401
2	59	-0.38	0.076432	0.084592	0.021267
12	48	-0.81	0.055224	0.070424	0.018663
10	50	-0.34	0.098897	0.108924	0.029715
10	53	-2.02	0.051419	0.091581	0.019920
9	54	-2.18	0.056668	0.118062	0.028152
14	48	-1.65	0.031761	0.062215	0.018475
18	44	-1.21	0.118284	0.161032	0.035246
17	45	-1.26	0.092759	0.134150	0.032728
19	43	-1.75	0.068818	0.118149	0.028166
10	49	-3.22	0.014295	0.063713	0.015346
0	59	-2.67	0.046856	0.098507	0.019356
18	41	-1.87	0.076901	0.135227	0.031238
3	56	-2.48	0.042513	0.078773	0.014632
2	57	-2.44	0.032513	0.078778	0.018934
10	48	-2.37	0.022315	0.071369	0.020673
18	40	-3.31	0.055472	0.171804	0.035126
15	43	-2.42	0.024695	0.085241	0.024988
19	39	-1.81	0.068696	0.121314	0.029015
12	46	-1.23	0.048397	0.066874	0.015012
0	58	-2.24	0.049298	0.103516	0.024159
9	49	-2.33	0.025405	0.058458	0.014184
17	41	-2.04	0.069026	0.127896	0.028850
9	48	-1.90	0.028653	0.066466	0.019934
2	56	-3.00	0.022407	0.068172	0.015232
17	40	-2.71	0.057658	0.148343	0.033454
18	39	-2.09	0.071061	0.137279	0.031751
8	49	-1.10	0.017324	0.032863	0.014159
15	42	-0.81	0.085805	0.106126	0.025228
2	55	-0.76	0.064417	0.083463	0.025139
10	47	-2.40	0.048509	0.119316	0.029447
19	38	-1.95	0.021008	0.038458	0.008930
3	54	-1.07	0.082127	0.112578	0.028434
16	41	-0.11	0.116602	0.119469	0.026871
1	56	-1.87	0.033367	0.065849	0.017354
16	40	-2.32	0.058308	0.132512	0.032046
17	39	-2.35	0.054736	0.124870	0.029858
14	42	-1.52	0.077791	0.120535	0.028206
10	46	-2.61	0.021357	0.064903	0.016677
9	47	-1.90	0.061402	0.113660	0.027508
1	55	-1.67	0.042887	0.090082	0.028229
3	53	-2.13	0.061547	0.101460	0.018754
18	38	-1.66	0.008596	0.024898	0.009842
2	54	-0.93	0.095343	0.123232	0.030070

15	41	-2.21	0.047438	0.094934	0.021535
18	45	-1.36	0.110735	0.155304	0.032860
1	62	-1.26	0.051573	0.082350	0.024457
4	52	-3.23	0.055025	0.109704	0.016946
15	48	-2.72	0.034698	0.091293	0.020808
17	46	-2.14	0.015522	0.052762	0.017390
0	63	-3.20	0.018233	0.087057	0.021505
16	46	-2.33	0.018915	0.059449	0.017364
15	47	-1.68	0.051187	0.094150	0.025583
9	53	-1.58	0.053011	0.083558	0.019296
2	60	-1.39	0.069385	0.108547	0.028094
18	43	-1.49	0.088287	0.133863	0.030602
15	46	-1.12	0.069910	0.088604	0.016739
3	58	-1.06	0.074582	0.103720	0.027538
15	45	-1.44	0.051337	0.092629	0.028638
14	46	-1.59	0.024930	0.049716	0.015545
2	58	-1.04	0.072580	0.100726	0.026949
11	49	-0.35	0.031205	0.036346	0.014565
18	42	-1.27	0.095702	0.136599	0.032190
13	47	-1.68	0.100805	0.157605	0.033815
5	55	-0.79	0.070640	0.091351	0.026301
1	59	-1.16	0.064467	0.093909	0.025319
7	53	-0.84	0.071615	0.088618	0.020159
6	54	-0.30	0.153610	0.163965	0.034462
9	51	-1.75	0.057024	0.101368	0.025381
3	57	-1.15	0.068258	0.089627	0.018660
18	50	-1.07	0.099407	0.132766	0.031123
2	66	-1.03	0.054096	0.076871	0.022195
17	51	-0.53	0.101202	0.116744	0.029452
12	56	-0.23	0.069793	0.072735	0.013022
8	60	-0.18	0.122816	0.128121	0.030188
4	58	-2.82	0.039148	0.101729	0.022166
10	52	-2.87	0.028455	0.093867	0.022769
12	49	-2.53	0.031699	0.066757	0.013843
19	42	-1.66	0.074324	0.123025	0.029417
14	47	-1.90	0.074535	0.131720	0.030051
0	61	-1.81	0.063673	0.102337	0.021329
9	52	-2.88	0.026002	0.086934	0.021129
10	59	-2.63	0.028501	0.086678	0.022144
17	52	-1.66	0.045269	0.084966	0.023905
3	66	-1.04	0.057710	0.081738	0.023185
5	64	-2.52	0.064155	0.145679	0.032340
1	68	-1.87	0.046375	0.100802	0.029105
9	60	-1.87	0.053877	0.103968	0.026821
0	70	-1.68	0.029325	0.072912	0.025944
3	67	-1.65	0.065167	0.108679	0.026309
9	61	-2.36	0.032153	0.086613	0.023056
17	53	-1.71	0.036969	0.074973	0.022281
16	54	-1.10	0.098640	0.132874	0.031234
19	51	-0.25	0.107243	0.114004	0.026951
6	64	-1.53	0.114360	0.167469	0.034793
2	68	-1.37	0.054620	0.089588	0.025474
10	60	-1.85	0.055616	0.108402	0.028580
0	71	-1.68	0.045835	0.087109	0.024521

10	61	-2.60	0.028224	0.092196	0.024607
17	54	-2.19	0.074748	0.146595	0.032820
3	68	-1.40	0.050732	0.087233	0.025996
7	64	-2.42	0.043527	0.111125	0.027967
19	45	-1.77	0.078566	0.133394	0.030993
2	62	-1.71	0.040812	0.077872	0.021734
18	46	-1.09	0.023132	0.041723	0.017106
1	63	-2.60	0.046426	0.124045	0.029806
9	55	-1.72	0.039430	0.080898	0.024155
12	52	-1.71	0.056721	0.089391	0.019093
10	54	-1.43	0.081567	0.124075	0.029809
0	64	-1.58	0.042768	0.081062	0.024279
17	48	-0.77	0.042790	0.060064	0.022312
7	58	-0.19	0.088919	0.094007	0.027082
16	49	-0.12	0.054843	0.056733	0.015785
17	47	-2.78	0.051851	0.140966	0.032082
4	60	-2.03	0.040157	0.084665	0.021979
2	63	-2.03	0.051618	0.103607	0.025638
18	47	-2.27	0.079017	0.157614	0.034619
10	55	-1.87	0.035528	0.084783	0.026372
4	61	-2.08	0.057725	0.097159	0.018959
1	64	-2.15	0.079515	0.154142	0.034687
5	60	-1.40	0.077780	0.121717	0.031377
18	48	-0.64	0.038844	0.052505	0.021210
3	63	-1.34	0.064793	0.098206	0.025020
14	52	-0.36	0.075506	0.083081	0.021117
12	54	-0.80	0.083919	0.104305	0.025374
1	65	-1.90	0.027378	0.070479	0.022726
17	49	-1.27	0.027721	0.047880	0.015912
19	47	-1.67	0.088462	0.140484	0.031144
11	55	-0.91	0.066133	0.089290	0.025465
19	48	-1.21	0.024680	0.051357	0.022100
17	50	-1.25	0.082105	0.120294	0.030550
13	54	-0.57	0.146856	0.166228	0.034184
16	51	-0.16	0.106684	0.111228	0.028479
7	60	-1.27	0.065891	0.098832	0.026010
13	63	-2.03	0.066504	0.125916	0.029211
18	58	-1.09	0.086702	0.118387	0.028992
15	61	-2.01	0.053552	0.098291	0.022227
17	59	-1.48	0.050982	0.086531	0.024061
10	66	-1.77	0.037272	0.079341	0.023743
12	65	-1.20	0.048230	0.069879	0.018084
19	58	-1.19	0.072692	0.105446	0.027465
18	59	-0.40	0.074809	0.084586	0.024620
11	67	-0.44	0.127350	0.140951	0.030849
15	63	-0.24	0.087190	0.092849	0.024065
17	61	0.62	0.108782	0.092730	0.026028
11	66	-1.73	0.035133	0.075059	0.023098
5	73	-1.68	0.093536	0.145454	0.030951
13	65	-0.80	0.040919	0.057222	0.020280
19	59	-0.92	0.059312	0.080169	0.022723
10	68	-2.11	0.035445	0.090461	0.026128
9	69	-1.46	0.088865	0.131885	0.029499
18	60	-1.66	0.090145	0.143744	0.032348

9	70	-1.95	0.070167	0.129409	0.030417
12	67	-1.21	0.074266	0.102978	0.023688
17	62	-1.00	0.051609	0.075126	0.023571
16	63	-0.59	0.094297	0.110675	0.027597
14	65	-1.13	0.042083	0.063968	0.019323
6	66	-1.29	0.048511	0.078467	0.023297
12	60	-1.29	0.062740	0.093995	0.024304
6	67	-0.51	0.135755	0.152737	0.033368
11	62	0.38	0.076602	0.068120	0.022234
8	65	-1.23	0.033626	0.058125	0.019963
15	64	-2.31	0.036696	0.096646	0.025990
10	69	-2.18	0.072492	0.140324	0.031055
19	60	-2.31	0.063070	0.130154	0.029016
1	71	-1.39	0.106643	0.155413	0.035160
2	70	-0.74	0.111164	0.134267	0.031161
0	72	-2.13	0.034525	0.087653	0.024893
5	67	-2.45	0.057717	0.133800	0.031089
7	66	-1.67	0.034903	0.073246	0.022957
19	54	-1.00	0.115060	0.146390	0.031451
13	60	-0.79	0.114866	0.141006	0.033050
15	60	-1.58	0.053278	0.093461	0.025432
12	73	-0.64	0.094835	0.110776	0.025009
15	70	-0.72	0.074916	0.095494	0.028566
19	68	-1.09	0.067836	0.096820	0.026483
14	72	-0.69	0.129775	0.152429	0.032820
16	67	-0.72	0.108322	0.129836	0.029754
14	68	-0.14	0.094271	0.097875	0.025332
19	73	-0.13	0.143054	0.147315	0.031758
18	71	0.18	0.173201	0.166714	0.035416
15	73	-0.37	0.097363	0.106339	0.024458
16	72	-0.03	0.146401	0.147466	0.033014
19	69	-0.20	0.158963	0.165811	0.034913
10	62	-2.73	0.018247	0.079916	0.022575
9	63	-1.87	0.053652	0.099749	0.024659
7	65	-2.31	0.021621	0.068633	0.020350
18	54	-1.60	0.109646	0.164689	0.034465
8	64	-1.59	0.102205	0.152909	0.031840
4	68	-1.56	0.047878	0.080858	0.021083
15	57	-2.67	0.053071	0.104889	0.019373
0	73	-2.43	0.034471	0.091736	0.023518
1	72	-2.25	0.086007	0.167596	0.036182
10	63	-2.39	0.043244	0.105606	0.026084
15	58	-2.32	0.042374	0.103368	0.026325
9	64	-2.43	0.049598	0.119705	0.028857
17	56	-1.82	0.026251	0.056086	0.016436
1	73	-1.84	0.094011	0.158907	0.035195
7	67	-1.23	0.078815	0.110237	0.025503
12	62	-1.39	0.050515	0.076850	0.018989
9	65	-2.01	0.028214	0.067155	0.019339
16	58	-1.60	0.060049	0.106602	0.029064
19	55	-1.51	0.049573	0.086199	0.024304
15	59	-1.55	0.058935	0.092105	0.021334
8	66	-1.69	0.036522	0.074513	0.022464
17	57	-1.42	0.038867	0.067988	0.020496

2	73	-1.26	0.094051	0.130727	0.029073
12	63	-1.03	0.068031	0.091572	0.022841
14	61	-0.49	0.079614	0.091183	0.023652
16	59	-0.77	0.068048	0.086059	0.023256
10	64	-2.75	0.044190	0.125845	0.029743
10	65	-1.97	0.028729	0.070983	0.021443
17	58	-1.86	0.055917	0.109998	0.029059
11	64	-1.07	0.117046	0.152103	0.032887
7	68	-0.24	0.075962	0.081899	0.024988
9	66	-2.32	0.024008	0.075280	0.022075
18	67	-2.63	0.068281	0.155922	0.033300
19	66	-2.32	0.018323	0.072891	0.023516
17	68	-1.56	0.057088	0.100123	0.027595
17	67	-2.17	0.075608	0.142051	0.030609
19	65	-1.56	0.029189	0.059554	0.019495
18	66	-1.69	0.038956	0.079362	0.023907
14	73	-1.62	0.090701	0.140362	0.030669
17	70	-1.16	0.124019	0.165563	0.035764
19	67	-1.75	0.087466	0.140535	0.030322
18	68	-1.54	0.065523	0.109424	0.028490
17	69	-0.76	0.141138	0.167805	0.035105
10	71	-2.50	0.053615	0.129062	0.030195
9	72	-1.74	0.078605	0.129387	0.029231
7	73	-0.90	0.095143	0.119087	0.026665
14	67	-2.37	0.059358	0.129962	0.029841
19	62	-1.44	0.037722	0.068623	0.021428
18	63	-1.92	0.069724	0.127177	0.029974
16	65	-1.71	0.031043	0.068370	0.021852
15	66	-2.02	0.039869	0.087138	0.023457
10	70	-1.79	0.077874	0.134985	0.031969
18	62	-1.24	0.041732	0.070588	0.023221
18	65	-1.01	0.040074	0.061268	0.020913
0	2	-5.27	0.047150	0.154877	0.020459
0	5	-4.41	0.036059	0.110101	0.016785
4	2	-4.44	0.061016	0.140584	0.017905
0	6	-4.24	0.045444	0.138232	0.021905
4	3	-4.00	0.026874	0.093593	0.016666
0	11	-4.53	0.019737	0.099056	0.017491
4	15	-4.34	0.071930	0.144213	0.016665
4	16	-5.12	0.041714	0.099519	0.011298
1	19	-4.18	0.014646	0.066606	0.012416
9	36	-4.42	0.019627	0.078231	0.013253
14	36	-3.67	0.013501	0.056649	0.011748
9	41	-2.75	0.039449	0.106173	0.024246
0	48	-4.15	0.021618	0.106259	0.020392
17	38	-3.94	0.011149	0.050017	0.009870
4	48	-3.48	0.036615	0.096893	0.017341
12	40	-2.72	0.029705	0.101630	0.026401
16	36	-3.19	0.026585	0.073373	0.014672
4	65	-3.80	0.029445	0.091961	0.016456
4	66	-3.52	0.021700	0.089011	0.019141
1	70	-2.60	0.068357	0.164769	0.037142
4	67	-1.86	0.045334	0.085057	0.021313
9	62	-1.88	0.035263	0.074886	0.021118

6	65	-1.10	0.036221	0.058852	0.020578
9	56	-3.36	0.017366	0.064887	0.014164
4	62	-3.24	0.041392	0.097515	0.017340
0	66	-3.39	0.022023	0.094156	0.021279
2	64	-2.42	0.053474	0.124628	0.029356
0	67	-2.43	0.033370	0.085231	0.021303
2	65	-1.56	0.037652	0.069294	0.020320
9	58	-1.58	0.057084	0.098164	0.025946
18	49	-0.81	0.021118	0.033548	0.015285
12	55	-0.78	0.060320	0.077568	0.022068
1	66	-1.69	0.034130	0.078739	0.026437
3	64	-1.01	0.083870	0.112901	0.028773
17	64	-2.83	0.054924	0.149706	0.033502
9	71	-2.35	0.055536	0.122006	0.028323
17	63	-2.26	0.053202	0.118432	0.028913
19	61	-1.38	0.053638	0.086904	0.024149
14	66	-1.01	0.056294	0.078365	0.021837
12	68	-0.12	0.077339	0.080066	0.022392
16	64	-1.77	0.079391	0.135779	0.031805
17	66	-1.42	0.043073	0.077880	0.024526
15	65	-2.89	0.029616	0.087850	0.020144
10	73	-2.66	0.053633	0.132397	0.029657
15	68	-1.73	0.040516	0.083611	0.024901
9	73	-1.32	0.087308	0.124163	0.027993
19	64	-2.91	0.054600	0.148196	0.032117
10	72	-1.98	0.074183	0.136062	0.031270
15	67	-1.48	0.062613	0.097532	0.023520
19	63	-0.74	0.095142	0.115738	0.027720
16	66	-1.01	0.054022	0.078714	0.024491
16	73	-1.08	0.106476	0.140635	0.031535
17	65	-2.38	0.014316	0.065516	0.021544
18	64	-2.53	0.079704	0.168551	0.035081
17	72	-1.54	0.108471	0.162129	0.034924
18	73	-0.59	0.144461	0.164796	0.034696
19	71	-0.00	0.147229	0.147386	0.032229
19	70	-2.08	0.094066	0.166315	0.034796
17	71	-1.39	0.103222	0.150916	0.034383
18	70	-1.61	0.132050	0.192246	0.037293
19	72	-1.58	0.106020	0.160429	0.034447
17	73	-1.85	0.090665	0.151402	0.032841
18	72	-1.53	0.125677	0.182295	0.037109
4	18	-5.46	0.038777	0.114486	0.013868
0	25	-4.94	0.025585	0.097763	0.014625
0	24	-5.46	0.026529	0.110699	0.015416
4	25	-5.02	0.019916	0.086820	0.013336
4	24	-4.84	0.029558	0.096958	0.013937
15	16	-4.49	0.033202	0.094249	0.013594
17	17	-4.19	0.016354	0.056870	0.009673
0	34	-4.09	0.021364	0.096663	0.018409
16	19	-4.08	0.025740	0.066995	0.010116
14	19	-4.49	0.011472	0.046244	0.007753
15	18	-4.56	0.037450	0.110726	0.016052
2	36	-4.20	0.026530	0.081551	0.013091
1	38	-4.74	0.019971	0.067935	0.010121

19	17	-3.92	0.012102	0.044685	0.008302
7	36	-4.69	0.018947	0.088027	0.014715
15	25	-4.50	0.017130	0.085860	0.015277
0	57	-4.86	0.035944	0.121383	0.017596
0	62	-3.99	0.028702	0.107088	0.019652
1	60	-3.00	0.049925	0.138741	0.029643
17	43	-2.22	0.049021	0.117039	0.030605
19	41	-2.56	0.052069	0.124190	0.028180
5	63	-1.68	0.067284	0.114389	0.028064
4	57	-3.78	0.049114	0.108204	0.015625
0	60	-2.95	0.021737	0.084521	0.021314
1	67	-2.20	0.082279	0.153144	0.032163
3	65	-2.29	0.027558	0.076598	0.021437
10	58	-2.27	0.039154	0.103398	0.028286
9	59	-2.58	0.027738	0.081452	0.020806
14	54	-1.66	0.087237	0.138051	0.030570
4	64	-1.60	0.046999	0.083796	0.023004
0	68	-2.88	0.016270	0.081315	0.022595
4	56	-4.88	0.043620	0.101217	0.011799
15	56	-4.53	0.032081	0.096509	0.014212
15	49	-4.18	0.026064	0.092269	0.015820
0	65	-3.90	0.027248	0.101362	0.018997
10	56	-3.57	0.014617	0.070055	0.015537
9	57	-3.10	0.018537	0.075041	0.018218
15	52	-3.50	0.038010	0.108953	0.020277
10	57	-3.16	0.020212	0.081746	0.019477
9	67	-3.21	0.028899	0.115155	0.026866
12	64	-2.49	0.040921	0.104866	0.025703
10	67	-2.90	0.041255	0.122344	0.027923
12	66	-1.96	0.034078	0.074365	0.020589
15	62	-3.00	0.032731	0.095685	0.020964
14	63	-2.21	0.051687	0.110681	0.026704
13	64	-2.06	0.098173	0.169524	0.034566
9	68	-1.61	0.047850	0.087111	0.024376
17	60	-1.80	0.074159	0.128747	0.030387
14	64	-2.44	0.063527	0.140490	0.031547
0	15	-6.47	0.042007	0.163191	0.018736
0	16	-6.96	0.031179	0.116832	0.012314
2	17	-6.58	0.022125	0.082685	0.009205
0	18	-6.67	0.028918	0.129025	0.015015
1	17	-6.32	0.017286	0.076173	0.009312
2	19	-6.24	0.018466	0.077070	0.009393
10	17	-5.69	0.028455	0.085512	0.010031
10	19	-6.18	0.020112	0.080462	0.009773
9	19	-6.25	0.016757	0.073479	0.009070
16	17	-5.32	0.017817	0.072630	0.010304
3	36	-6.12	0.024578	0.101177	0.012517
2	38	-6.11	0.016611	0.072523	0.009145
0	46	-5.45	0.025519	0.106874	0.014936
10	36	-4.76	0.021536	0.085232	0.013384
7	38	-6.20	0.023686	0.081561	0.009328
3	17	-7.27	0.031382	0.102580	0.009788
0	20	-7.45	0.030902	0.150976	0.016116
7	19	-7.00	0.019804	0.086810	0.009577

9	17	-6.52	0.021411	0.079279	0.008870
3	38	-6.89	0.019125	0.090142	0.010305
9	38	-6.07	0.015755	0.069140	0.008800
15	36	-6.48	0.033633	0.127962	0.014565
0	49	-5.77	0.030233	0.113813	0.014476
12	36	-5.49	0.019619	0.091717	0.013133
10	38	-5.45	0.020811	0.075512	0.010045
16	38	-5.04	0.015525	0.063851	0.009591
4	49	-5.37	0.029125	0.098596	0.012932
0	53	-4.90	0.035530	0.133033	0.019896
0	52	-4.83	0.026133	0.119843	0.019383
0	56	-6.31	0.030776	0.114803	0.013315
3	19	-8.05	0.024332	0.098375	0.009201
7	17	-8.05	0.022728	0.092735	0.008692
12	19	-8.18	0.020534	0.090402	0.008537
12	38	-7.57	0.018599	0.084238	0.008669
12	17	-9.31	0.023398	0.096220	0.007825
0	36	-8.74	0.034375	0.159845	0.014356
4	36	-8.25	0.036506	0.139220	0.012452
15	38	-8.49	0.031789	0.121523	0.010574
15	19	-10.94	0.028951	0.132179	0.009439
4	38	-11.36	0.035412	0.131769	0.008478
15	17	-12.58	0.030879	0.137731	0.008491
0	38	-12.88	0.034941	0.155284	0.009343
0	19	-14.16	0.036927	0.169821	0.009388
4	19	-14.14	0.031627	0.144835	0.008008
0	17	-18.14	0.038079	0.174829	0.007538
4	17	-17.42	0.033349	0.149877	0.006689
