# Supplementary figures and images for: Identification of Position-Specific Correlations between DNA-Binding Domains and Their Binding Sites. Application to the MerR Family of Transcription Factors
Source: PLoS One. 2016 Sep 30;11(9):e0162681. doi: 10.1371/journal.pone.0162681 (PMC5045206; doi:10.1371/journal.pone.0162681)

### Supporting Figure S1. B-cutoff plot.

Global minimum p-value corresponds to 32 pairs.

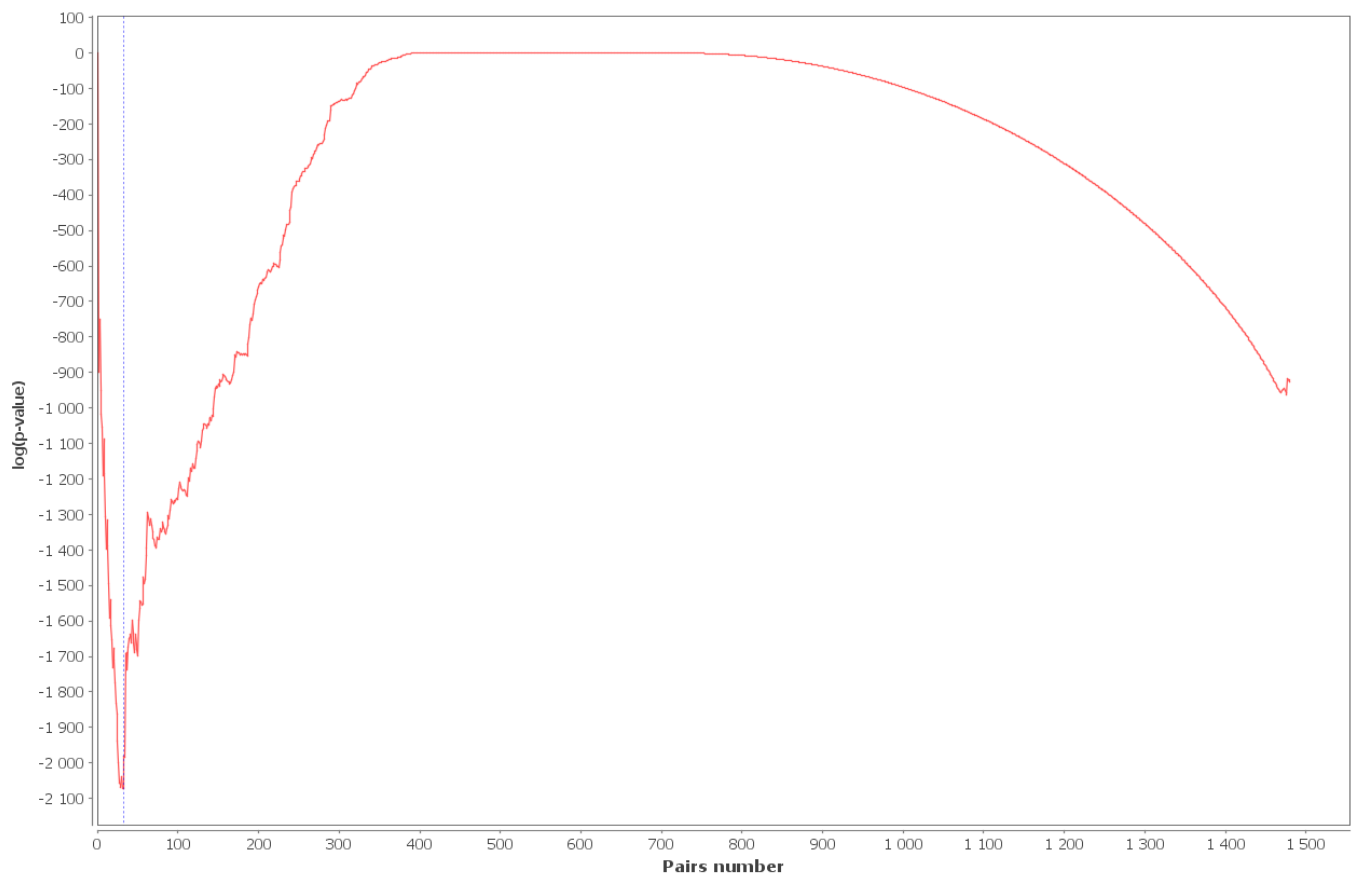

Supplement: S1 Fig — Global minimum p-value corresponds to 32 pairs. (PDF) [file pone.0162681.s007.pdf]

Supporting Figure S7. B-cutoff plot for shuffled regulator-site pairs

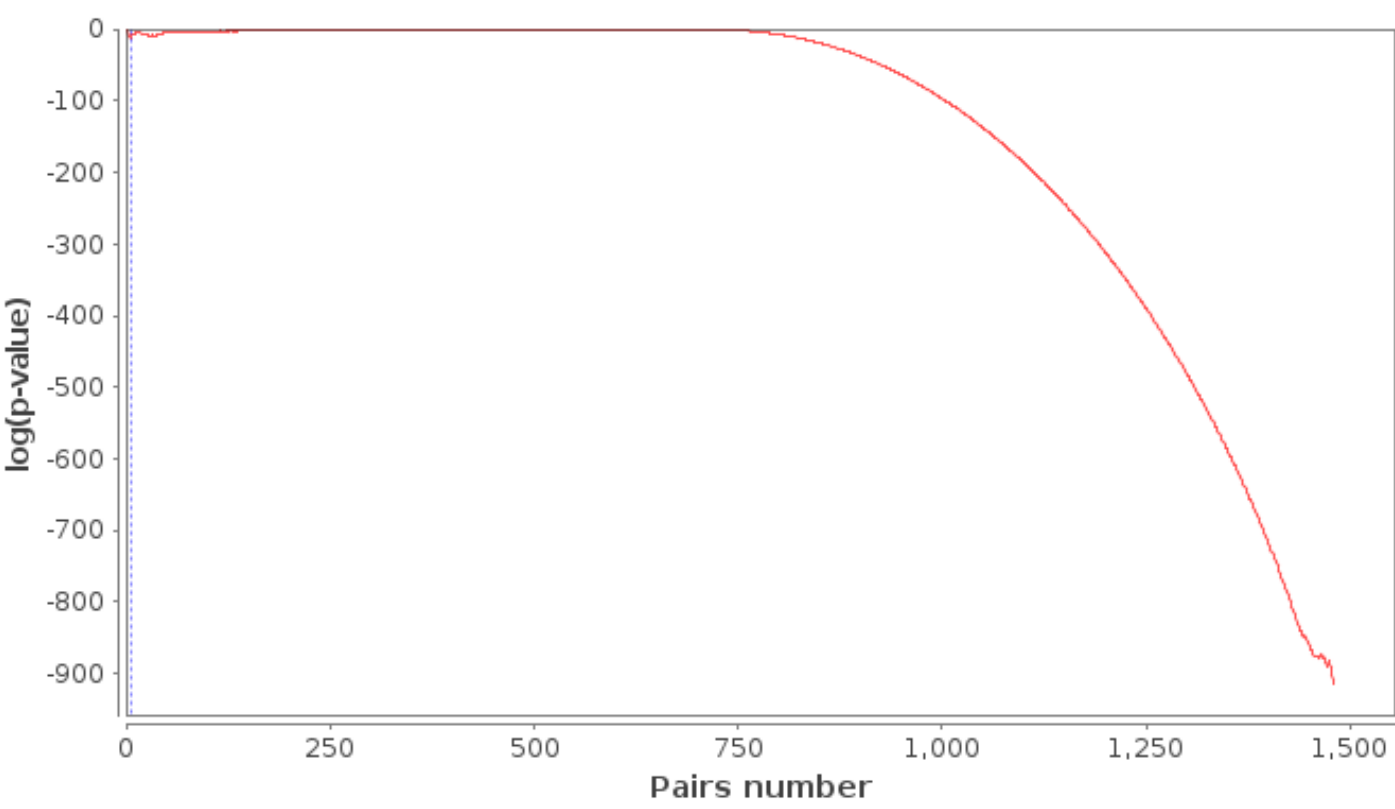

Supplement: S7 Fig — (PDF) [file pone.0162681.s013.pdf]
