# Supplementary material for: Identification of Position-Specific Correlations between DNA-Binding Domains and Their Binding Sites. Application to the MerR Family of Transcription Factors
Source: PLoS One. 2016 Sep 30;11(9):e0162681. doi: 10.1371/journal.pone.0162681 (PMC5045206; doi:10.1371/journal.pone.0162681)

Supporting figure S2. Heatmap of protein-DNA correlations with complete map of contacts.

TF positions are along the horizontal axis and at the Logo above. Site positions are along the vertical axis and at Logo on the left. The color denotes the Z-score for a pair of positions with the color palette for significantly correlated pairs in the yellow to red interval, while black through light green colors denote positions below the significance threshold. Protein-DNA interactions are shown as stars. Interactions observed in the structures of complexes at least once are shown. Elements of protein secondary structure (from the crystal structure of E. coli CueR – PDB ID 1Q05) are shown at the top.

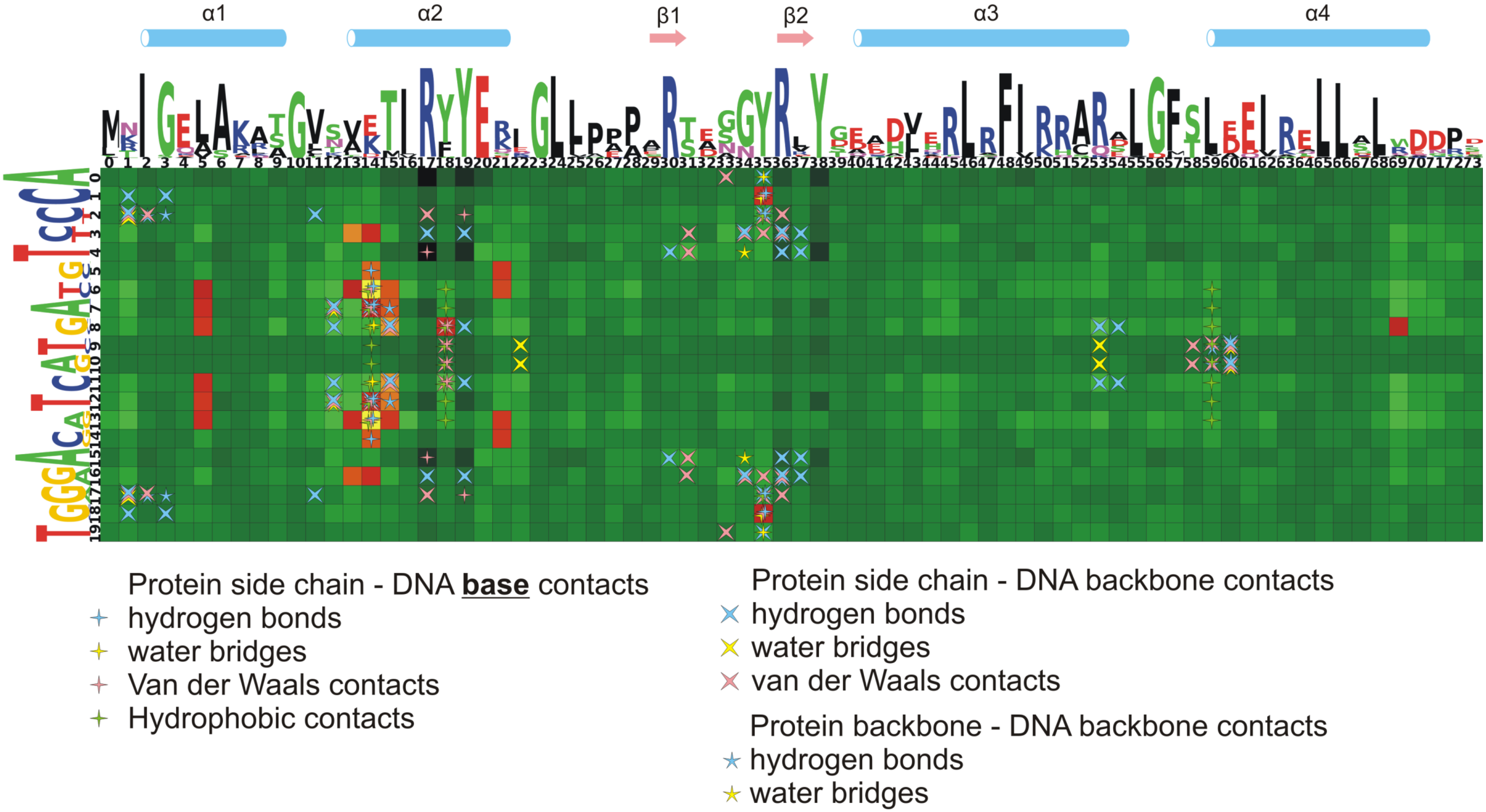

Supplement: S2 Fig — TF positions are along the horizontal axis and at the Logo above. Site positions are along the vertical axis and at Logo on the left. The color denotes the Z-score for a pair of positions with the color palette for significantly correlated pairs in the yellow to red interval, while black through light green colors denote positions below the significance threshold. Protein-DNA interactions are shown as stars. Interactions observed in the structures of complexes at least once are shown. Elements of protein secondary structure (from the crystal structure of E. coli CueR – PDB ID 1Q05) are shown at the top. (PDF) [file pone.0162681.s008.pdf]
