# Supplementary material for: Identification of Position-Specific Correlations between DNA-Binding Domains and Their Binding Sites. Application to the MerR Family of Transcription Factors
Source: PLoS One. 2016 Sep 30;11(9):e0162681. doi: 10.1371/journal.pone.0162681 (PMC5045206; doi:10.1371/journal.pone.0162681)

Supporting figure S6. Phylogenetic tree of the TFs from the MERR family with pairs of residues in positions [6,21].

Colors of branches show overrepresented pairs of residues in positions [6,21] (see color code in the picture). Background colors show TF subfamilies: red – CUER, blue – MERR, green – CADR-PBRR, beige – CADR-PBRR-like, pink-HMRTR.

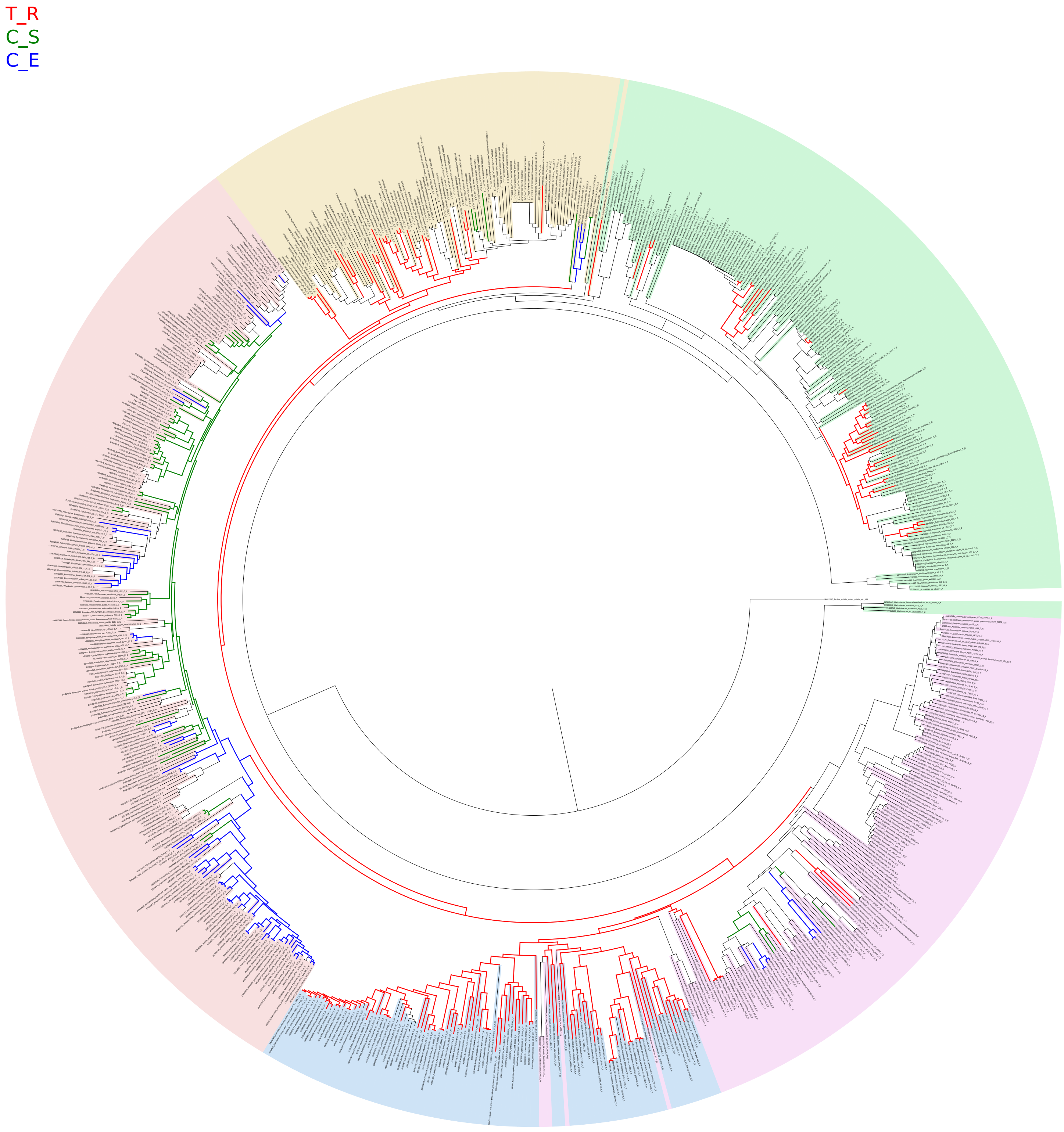

Supplement: S6 Fig — Colors of branches show overrepresented pairs of residues in positions (6,21) (see color code in the picture). Background colors show TF subfamilies: red—CueR, blue – MerR, green—CadR-PbrR, beige—CadR-PbrR-like, pink—HMRTR. (PDF) [file pone.0162681.s012.pdf]
